# Simulating Expansion of the Intracranial Space to Accommodate Brain Swelling after Decompressive Craniectomy: Volumetric Quantification in a 3D CAD Skull Model with Contour Elevation

**DOI:** 10.3390/brainsci11040428

**Published:** 2021-03-27

**Authors:** Woon-Man Kung, Yao-Chin Wang, I-Shiang Tzeng, Yu-Te Chen, Muh-Shi Lin

**Affiliations:** 1 Department of Exercise and Health Promotion, College of Kinesiology and Health, Chinese Culture University, Taipei 11114, Taiwan; nskungwm@yahoo.com.tw (W.-M.K.); istzeng@gmail.com (I.-S.T.); 2 Graduate Institute of Injury Prevention and Control, College of Public Health, Taipei Medical University, Taipei 11031, Taiwan; vkwang8888@yahoo.com.tw; 3 Department of Emergency, Min-Sheng General Hospital, Taoyuan 33044, Taiwan; 4 Institute of Applied Mathematics, College of Science, National Cheng Kung University, Tainan 70101, Taiwan; yute8368@gmail.com; 5 Division of Neurosurgery, Department of Surgery, Kuang Tien General Hospital, Taichung 43303, Taiwan; 6 Department of Biotechnology and Animal Science, College of Bioresources, National Ilan University, Yilan 26047, Taiwan; 7 Department of Biotechnology, College of Medical and Health Care, Hung Kuang University, Taichung 43302, Taiwan; 8 Department of Health Business Administration, College of Medical and Health Care, Hung Kuang University, Taichung 43302, Taiwan

**Keywords:** decompressive craniectomy, 3D CAD skull model, image processing, quantitative analysis, numerical integration with the rectangle method

## Abstract

*Background*: Decompressive craniectomy (DC) can be used to augment intracranial space and halt brainstem compromise. However, a widely adopted recommendation for optimal surgical extent of the DC procedure is lacking. In the current study, we utilized three-dimensional (3D) computer-assisted design (CAD) skull models with defect contour elevation for quantitative assessment. *Methods*: DC was performed for 15 consecutive patients, and 3D CAD models of defective skulls with contour elevations (0–50 mm) were reconstructed using commercial software. Quantitative assessments were conducted in these CAD subjects to analyze the effects of volumetric augmentation when elevating the length of the contour and the skull defect size. The final positive results were mathematically verified using a computerized system for numerical integration with the rectangle method. *Results*: Defect areas of the skull CAD models ranged from 55.7–168.8 cm^2^, with a mean of 132.3 ± 29.7 cm^2^. As the contour was elevated outward for 6 mm or above, statistical significance was detected in the volume and the volume-increasing rate, when compared to the results obtained from the regular CAD model. The volume and the volume-increasing rate increased by 3.665 cm^3^, 0.285% (*p* < 0.001) per 1 mm of contour elevation), and 0.034% (*p* < 0.001) per 1 cm^2^ of increase of defect area, respectively. Moreover, a 1 mm elevation of the contour in Groups 2 (defect area 125–150 cm^2^) and 3 (defect area >150 cm^2^, as a proxy for an extremely large skull defect) was shown to augment the volume and the volume-increasing rate by 1.553 cm^3^, 0.101% (*p* < 0.001) and 1.126 cm^3^, 0.072% (*p* < 0.001), respectively, when compared to those in Group 1 (defect area <125 cm^2^). The volumetric augmentation achieved by contour elevation for an extremely large skull defect was smaller than that achieved for a large skull defect. *Conclusions*: The 3D CAD skull model contour elevation method can be effectively used to simulate the extent of a space-occupying swollen brain and to quantitatively assess the extent of brainstem protection in terms of volume augmentation and volume-increasing rate following DC. As the tangential diameter (representing the degree of DC) exceeded the plateau value, volumetric augmentation was attenuated. However, an increasing volumetric augmentation was detected before the plateau value was reached.

## 1. Introduction

Under emergent situations such as traumatic brain injury (TBI) and massive ischemic stroke, decompressive craniectomy (DC) is performed as an effective surgical strategy to open the dura and remove part of the skull, which helps to augment the intracranial space. This facilitates the outward expansion of the injury-driven swollen brain, thereby preventing the compromise of the brainstem [1,2]. DC has been shown to attenuate intracranial pressure (ICP) levels, as well as increase blood flow and tissue oxygen tension in the brain, consequently improving long-term outcomes [1,3,4,5]. However, little is known in the literature about the required size of the surgical resection for the skull bone. Subjective suggestions have been addressed individually, such as at least 60 cm^2^ of cranial bone for the DC procedure [3,5]. Guidelines which may be widely adopted for details of the DC procedure, such as elevation height of the scalp and size of the skull-cutting, are warranted in clinical management and research.

Advancements in CAD/CAM (computer-assisted design/manufacturing) techniques and progressive 3D (three-dimensional) printing make it feasible to create an accurate 3D CAD reconstruction of the skull [6,7,8]. Pathological conditions such as congenital heart defects [9], fractured pelvis [10], and organ bioprinting for tissue engineering [11] can be simulated using 3D printed models for surgical training and education, which can help advance the field of medicine. We previously demonstrated a methodology to design an algorithm for regular CAD models using the Open Source Computer Vision (OpenCV) and Open Graphic Library (OpenGL) OBJ Viewer. These proposed 3D CAD reconstructions with a regular contour, in which the curvature is symmetrical to the opposite side of the skull, were statistically verified to be greater than 95% and identical to 99.5%, indicating highly accurate reconstruction by CIS (cranial index of symmetry) scores [12].

In the current research, using the established symmetrically regular CAD/CAM reconstruction, algorithms of a CAD/CAM technique to reconstruct elevating contours were applied to simulate the clinical situation of injury-driven brain swelling. This clinical condition may cause the scalp to bulge outward from the skull and is a defect observed in patients who undergo the DC procedure. The overall objective of this study was to determine the potential optimal height for scalp elevation and the area of the skull defect, to obtain the major extent of volume expansion in CAD/CAM designs. Positive findings of the current quantitative analysis in 3D CAD reconstruction may provide clinicians with operative suggestions for the DC procedure to effectively achieve brain volume expansion and, consequently, to attenuate a fatal brainstem compromise in clinical practice.

## 2. Materials and Methods

### 2.1. Patients

From September 2013 to June 2014, 15 consecutive patients with either intracranial hematomas or malignant brain edemas caused by massive ischemic strokes were surgically treated. All 15 patients presented with marked neurological deficits and underwent cranial decompressive surgery. The operative method included a wide DC alone (for malignant cerebral infarction) (*n* = 3) and a wide DC combined with the removal of a hematoma on the lesioned side (*n* = 12). We reviewed the computed tomography (CT) scans of these patients with skull defects. The current study was performed in accordance with the Declaration of Helsinki and was approved by Institutional Review Board of the Taipei City Hospital (TCHIRB-1020807-E).

### 2.2. Clinical Cases before and after DC

**Figure 1:**
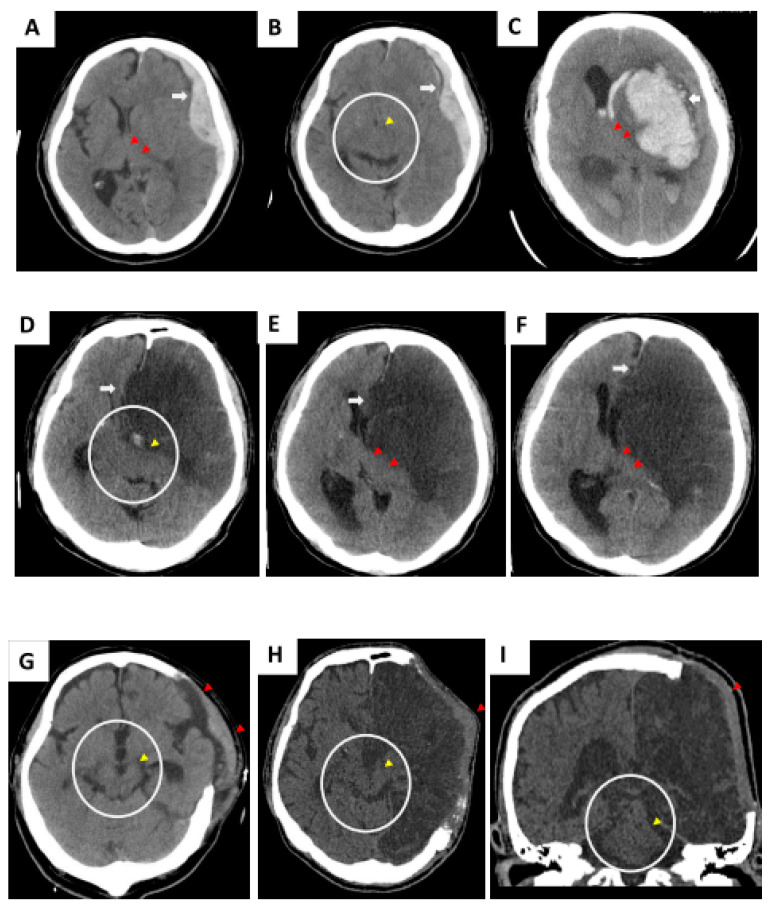
Initial computed tomography (CT) scan showing compromised brainstem due to acute subdural hematoma (SDH) (**A**,**B**, case 1), hemorrhagic stroke (**C**, case 2), and malignant cerebral infarction (**D**–**F**, case 3). Alteration of brainstem shape was caused by intracranial mass effect. Wide decompressive craniectomy (DC) should be performed immediately to create extensive volume, which accommodates postoperative brain swelling. (**G**–**I**) Brainstem shape was morphologically regained.

Representative pathological lesions of clinical cases 1–3 are highlighted in Figure 1A,B (TBI: acute subdural hematoma), Figure 1C (hemorrhagic stroke), and Figure 1D–F (malignant infarction-driven brain edema) respectively. Wide DC is beneficial to create additional volume for the injured brain to swell outward (indicated as a red triangle in Figure 1G (case 1), Figure 1H (case 3), and Figure 1I (case 3)) and, consequently, vacate a space for brainstem restoration through the removal of part of the skull bone together with the release of tension from the dura mater. After the DC procedure, the brainstem was morphologically restored, and the ambient cistern was opened widely (yellow arrow in circled area, Figure 1G (case 1), Figure 1H (case 3), and Figure 1I (case 3)).

### 2.3. Computed Tomography

CT JPEG images from a PACS system (GE PACS Web System) were analyzed in the current study. All fields of view of the images were 30 cm in diameter. The matrix size of the CT images was 512 × 512. Patients received CT scans of the brain with a slice thickness of 1.25 mm from the foramen magnum to the vertex of the skull, including the region of the skull defect. The thin-sliced high-resolution CT images were prepared for the 3D reconstruction of the skull [4,12].

### 2.4. Regular 3D CAD Reconstruction of the Skull Defects

An algorithm for 3D CAD reconstructions with a regular contour, of which the curvature is symmetrical to the opposite side of the skull, was previously demonstrated using OpenCV and OpenGL OBJ Viewer [12]. For editing two-dimensional (2D) images of brain CT scans, the steps were as follows: (1) load 2D images of the CT bone window (with skull defects) in a JPEG file format with OpenCV, for reconstruction; (2) appoint the axis of symmetry of skull in the CT bone window image according to the relative bony landmark in the midline; (3) the system fills the defect by creating a mirror-reflection of the contralateral skull curvature based on the axis of symmetry; (4) designate the upper and lower boundaries of the reconstructed contour to precisely match that of the skull defect. By repeating the aforementioned steps for all slices of the CT bone window images, the system was able to generate all the contours of the skull defect for all CT scan slices.

### 2.5. Reconstructing the Elevated Contour for the Skull Defects on 2D Images of Brain CT Scans Using OpenCV

**Figure 2:**
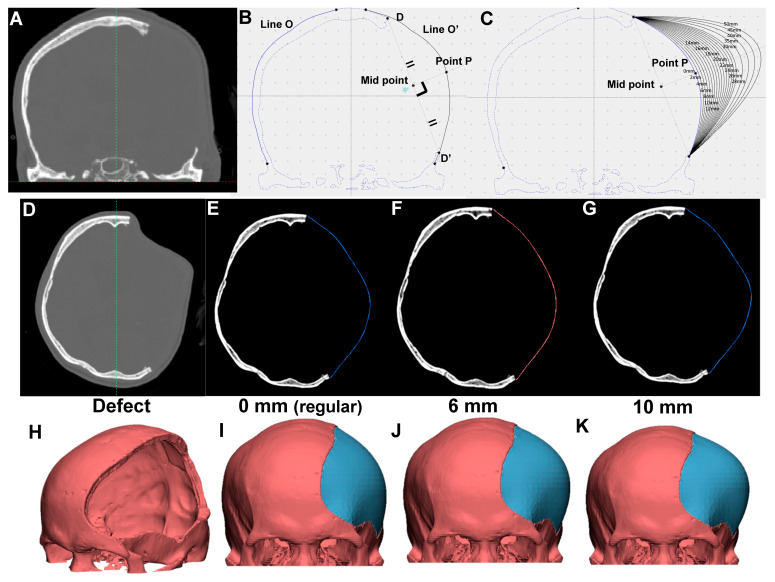
Three-dimensional (3D) computer-assisted design (CAD) model for skull defect reconstruction of case 3 using OpenCV and OpenGL OBJ Viewer. (**A**) CT bone window image with left skull defect. (**B**) Schematic illustration of symmetric (regular) contour planning. (**C**) Schematic illustration of elevated contour of skull defect. Contours elevated outward every 2 mm up to 30 mm, and every 5 mm up to 50 mm. Reconstructed contour from skull defect (**D**), with symmetrically filled (**E**), 6 mm (**F**), and 10 mm (**G**) contour elevation (**H**–**K**) with respective 3D model shown using OpenGL OBJ Viewer. Reconstructed artificial flap is labeled in blue.

Figure 2A shows an example of a CT bone window image with a skull defect and a schematic illustration of the proposed reconstructed contour planning (Figure 2B,C) for case 3. Image-editing software allows the user to change the position of the axis of symmetry and display a preview of the axis with the CT bone window image. The designated axis of symmetry (the green line) on the CT bone window image is shown in Figure 2A.

As shown in Figure 2B, the two projection points of the skull defect (point D and D′) are connected, and a perpendicular bisector is made through the connection of the two points. The symmetric contour for the skull defect (line O″) is generated according to the contralateral skull curvature (line O) based on the axis of symmetry. The intersection of the O′ line and the perpendicular bisector is point P. Assuming that point P is shifted outward by X mm along the perpendicular bisector through point P, the intersection with the perpendicular bisector is point X. We defined the proposed “elevated contour” as the arc line connecting points D, D′, and X. As indicated in Figure 2C, the reconstructed contours of the skull defect are elevated outward for every 2 mm up to 30 mm, and then for every 5 mm up to 50 mm for all 15 models.

These contours for the skull defects in 2D CT images were subsequently reconstructed for 3D CAD models using OpenGL OBJ Viewer. Specifically, 3D CAD reconstructions with contour elevation were used to simulate clinical conditions, mimicking the outward swelling of the injured brain and tissues from the DC-driven skull defect. For example, the original skull defect, regular contour, and the reconstruction of skull defect with 6 mm and 10 mm elevations for case 5 are shown as Figure 2D, Figure 2E, Figure 2F, and Figure 2G respectively. The corresponding 3D models are presented in Figure 2H–K. The reconstructed artificial flap is labeled in blue.

### 2.6. Reconstructing 3D Models Using OpenGL OBJ Viewer

**Figure 3:**
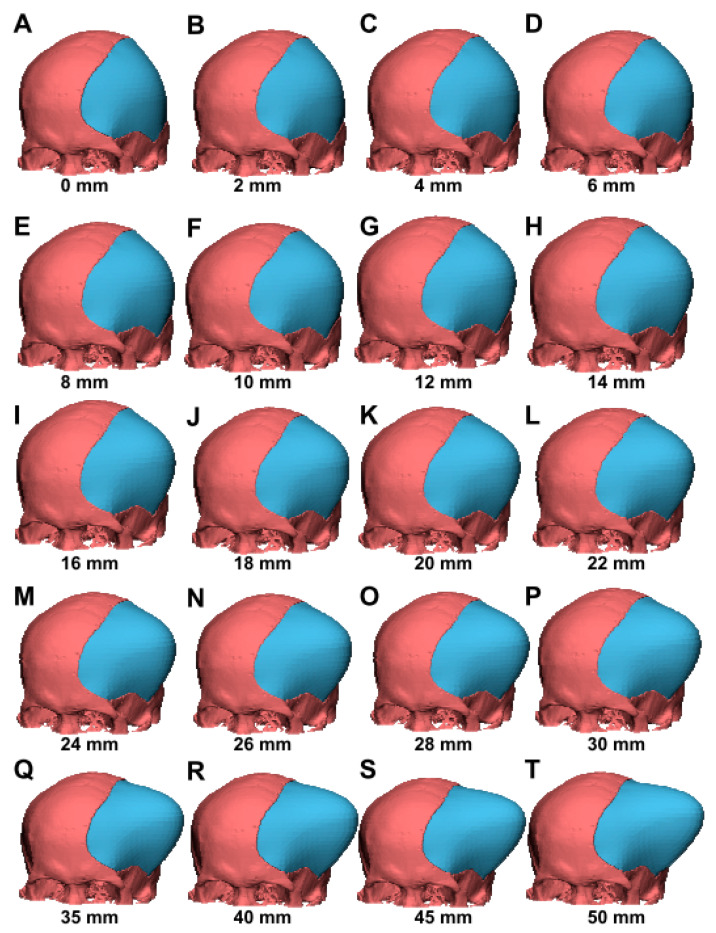
Various 3D CAD models with an elevated contour from 2–50 mm (case 3). The CAD model included algorithms for contour elevation, mimicking scalp outward bulging and, therefore, creating additional extracranial space for accommodation of brain swelling. (**A**–**T**) The 2–50 mm contour elevations represent various extents of volume expansion.

The algorithms used to build the 3D model for the reconstruction of the skull defect were described in our previous publication [12]. The software we used utilizes OpenGL OBJ Viewer to demonstrate the 3D model. In brief, the software initially extracts points surrounding the contour in each of the CT bone window images and further connects the adjacent points to form triangles. Assuming that there are *N_L_* surrounding points in image *L,* and *N_L_*_+1_ surrounding points in image *L* + 1, the adjacent point in image *L +* 1 of the *m-*th point in image *L* is the (m * N_L+__1_*/N_L_)-*th point in image *L* + 1. According to the following equation: let *n =* (m * N_L__+1_*/N_L_*), for the *m-*th point in image *L*, the software constructs one triangle by connecting the *m-*th and (*m* + 1)-th points in image *L,* and the *n-*th point in image *L* + 1, and another triangle by connecting the (*m +* 1)-th point in image *L* and the *n-*th and (*n* + 1)-th points in image *L* + 1. By forming two triangles for each point in each image, the software generates several triangles which surround the surface of the skull. Figure 3 shows an example of the 3D model with a 2–50 mm contour elevation for case 3. To discriminate the original bone from the newly reconstructed bone, the original bone is colored in red and the newly reconstructed bone is colored in blue. The generated 3D model is presented in an OBJ file format.

### 2.7. Computer-Assisted Quantitative Analysis for the Surface Area of the Skull Defect and Brain Tissue Volume

To the software platform (trial version of Materialise 3-matic), 15 3D model objects were loaded in an OBJ file format. For the measurements of the surface area of the skull defect, the user can rotate the 3D model into a true lateral view. The contour curve for the margin of the skull defect was outlined. A sagittal plane crossing the maximal region of the skull defect was defined. The projection of the defect margin to the sagittal plane comprised the surface area of the skull defect. With the use of a software tool, the defect area was calculated and presented as square millimeters. 

**Figure 4:**
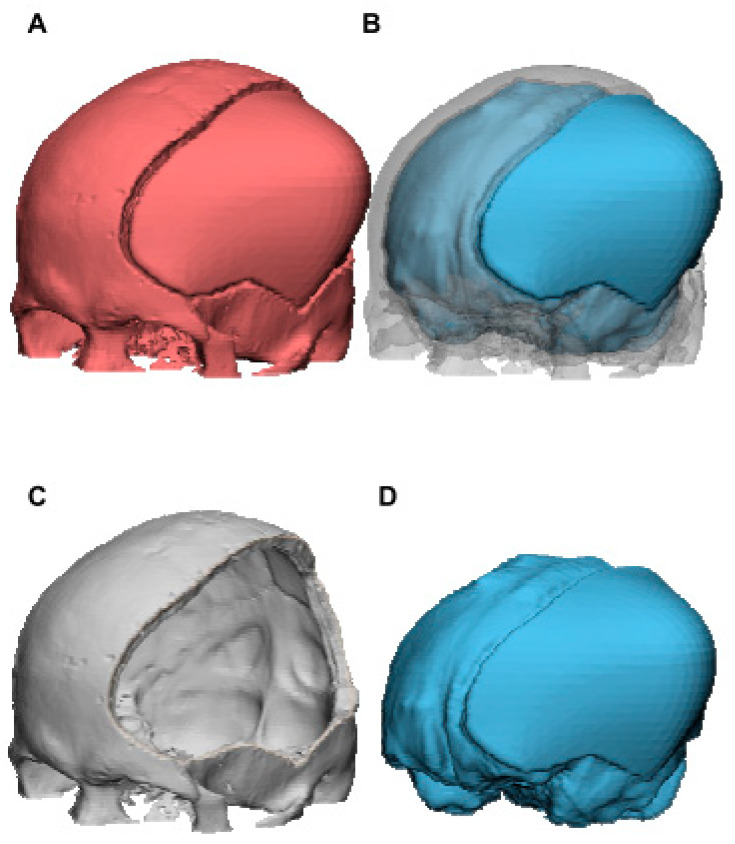
The 3D CAD model for calculation of brain tissue volume of case 3 using Materialise 3-matic. Brain tissue volume (**D**) was obtained by subtracting those of skull bone (**C**) from the whole CAD reconstruction (**A**). Volume of (**B**), which corresponded to (**A**), is equal to the sum of (**C**,**D**).

The following calculation was performed to obtain the brain tissue volume for the 15 subjects: volumes from the whole CAD reconstruction (red color, Figure 4A), which corresponded to the two-color mixing model of Figure 4B (skull bone in gray color plus brain tissue in blue color), minus those of skull bone (indicated in gray color, Figure 4C), are equal to the brain tissue volume (indicated in blue color, Figure 4D). Brain tissue volume and volume-increasing rate were calculated for each outward expansion in each model.

### 2.8. Ellipsoid Model of the Skull Following DC Procedure

The elliptical mathematical skull model was used to verify the relationship between skull defect sizes (a proxy of the extent of wide DC) and the corresponding efficacy in volumetric augmentation. If we consider the skull to be an ellipsoid, the following Equation (1) for an ellipsoid can be applied:

(1) $$\frac{x^{2}}{a^{2}}+\frac{y^{2}}{b^{2}}+\frac{z^{2}}{c^{2}}=1,$$

where *a* is the distance centrally to laterally, *b* is the distance centrally to anteriorly/posteriorly, and *c* is the distance centrally to superiorly/inferiorly (supposing that the center of symmetry at the ellipsoid represents the center point of the skull).

**Figure 5:**
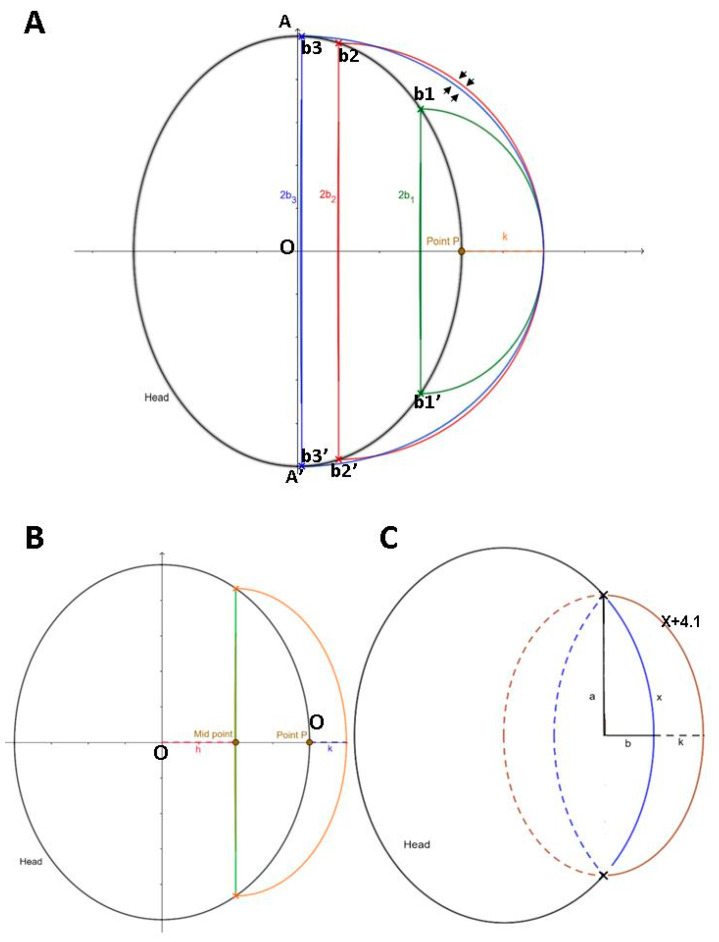
Schematic diagrams of ellipses as a proxy of skull following DC procedure. (**A**) When comparing the large (red) with extreme large (blue) extent of DC, the newly increased area under red contour is larger than that of blue contour (indicated by arrows). (**B**) Schematic diagram of ellipse with a part of defect (a proxy of defective skull) and newly created curve (K = height of contour elevation), as with the same setting of contour planning (as shown in Figure 2B,C). Analysis of calculus and numerical integration with rectangle method was undertaken according to the proposed schematic diagram. (**C**) Generalized mathematical formula for length of scalp elevation. Blue arch = regular contour (X: arc length). Length of orange arch = maximal length extension of blue arc = X + 4.1 (K = maximal height of contour elevation). a = length of the major axis for blue and orange ellipse. Lengths of the minor axis of blue and orange ellipses are b and b + k, respectively. Maximal lengths of a and b are approximately equal to the distance from head center to the most anterior and lateral margin. K = 16.4a + (8.2π − 16.4) × b/X(2π − 4) = (16.4a + 9.348b)/2.28X; let π = 3.14.

A schematic representation of the cross-section (axial view) of the CT image in a proposed patient following DC is shown in Figure 5A. Let the axial surface cross the center point (point O), i.e., c = 0 in the ellipsoid equation.

The outline of the black ellipse can be regarded as the axial section of the skull cross center, where AA′ is the major axis along the *Y*-axis of the ellipse and its length = 2A. Vertical to the *X*-axis, we assume three sets of intercept points along the outline of the black ellipse, which represent different sizes of skull defects resulting from a DC (smaller extent of DC (skull defect margin *b*_1_*b*_1_′); the largest extent of DC, which almost crosses the midline of the black ellipse (skull defect margin *b*_3_*b*_3_′); the second largest extent of DC (close to *b*_3_*b*_3_′ DC with a skull defect margin *b*_2_*b*_2_′)). According to the principle of mirror reflection of the design for 2D contour/3D CAD modeling, the three sets of the skull defect (intercept points *b*_1_*b*_1_′, *b*_2_*b*_2_′, and *b*_3_*b*_3_′) share the same P point for 2D contours, and K is set as the contour elevation height.

### 2.9. Determination of Parameters for Quantitation in Computerized Numerical Integration

Figure 5B shows a schematic diagram of an ellipse representing the skull defect following a DC procedure. Let the axial section of the skull be described by the following Equation (2):

(2) $$\frac{x^{2}}{a_{2}^{2}}+\frac{y^{2}}{b_{2}^{2}}=1.$$

The diameter of the tangent is represented by the green line segment (a proxy of the skull defect margin). The distance from the skull center (point O) to the tangent is h. By means of mirror reflection, a symmetrically regular contour is established (the black arc outside the green tangent), and the point P is the intersection of this arc on the *X*-axis. From point P, an elevation of a contour with height k (mm) (orange arc) is made to form a new area of the head (the area enclosed between the orange arc and the black arc). Let the midpoint (the intersection of the tangent on the *X*-axis) be the center of the new ellipse (where *a_h_*,*b_h_* are the semi-minor axis and the semi-major axis of the new ellipse, respectively). The ellipse Formula (3) is as follows:

(3) $$\frac{{\left(x-h\right)}^{2}}{a_{h}^{2}}+\frac{y^{2}}{b_{h}^{2}}=1.$$

Firstly, we calculate the new area in the first quadrant. The formula of the ellipse, $\frac{x^{2}}{a_{2}^{2}}+\frac{y^{2}}{b_{2}^{2}}=1,$ can be rearranged as $y=b{\left(1-\frac{x^{2}}{a^{2}}\right)}^{\frac{1}{2}}$. From Figure 5B, we can obtain $a_{h}=a+k-h$, and $b_{h}=b{\left(1-\frac{h^{2}}{a^{2}}\right)}^{\frac{1}{2}}$. Then, let the point on the new ellipse be $\left(x_{h},y_{h}\right)$, and rearrange the abovementioned ellipse formula to derive the following equation: $y_{h}=b{\left(1-\frac{x^{2}}{a^{2}}\right)}^{\frac{1}{2}}\times {\left[1-\frac{{\left(x-h\right)}^{2}}{{\left(a+k-h\right)}^{2}}\right]}^{\frac{1}{2}}$.

Using the Riemann integral, we can obtain the formula for calculating the increase in area (∆A), i.e., $\Delta A=\int_{a}^{a+k}y_{h}dx+\int_{h}^{a}\left(y_{h}-y\right)dx$.

Next, we use the rectangle method of numerical integration, whereby x can be subdivided into n equal parts in the two definite integrals, as follows: $\left\{\overset{n-1}{\underset{i=0}{\sum }}\frac{k}{n}{\times y}_{h}\left(x_{i}\right)+\overset{n}{\underset{i=1}{\sum }}\frac{a-h}{n}\times \left[y_{h}\left(x_{i}\right)-y\left(x_{i}\right)\right]\right\}$.

Since this is only the upper half of the first quadrant, the overall newly increased area is two times that of the above, i.e., $2\times \{\overset{n-1}{\underset{i=0}{\sum }}\frac{k}{n}{\times y}_{h}\left(x_{i}\right)+\overset{n}{\underset{i=1}{\sum }}\frac{a-h}{n}\times \left[y_{h}\left(x_{i}\right)-y\left(x_{i}\right)\right]\}$.

The formula for the volume-increasing rate is as follows: $\frac{\mathrm{Newly}\;\mathrm{increased}\;\mathrm{area}}{\mathrm{Original}\;\mathrm{total}\;\mathrm{area}}=\frac{2\times \Delta A}{4\times \int_{0}^{a}\mathrm{ydx}}=\frac{\Delta A}{2\times \sum_{i=0}^{n-1}\frac{a}{n}\times y(x_{i})}\times 100\%$.

### 2.10. Computerized System for Numerical Integration with the Rectangle Method Using the Visual Basic Programming Language

To simplify the evaluation of definite integral, Visual Studio 2017 (coded in the C programming language) was used. Data graphics were further obtained using MATLAB R2016a. In the code, *y* stands for the newly increased area, while *y*1, *y*2, *y*3, and *y*r refer to $\int_{a}^{a+k}y_{h}dx$, $\int_{h}^{a}(y_{h}-y)dx$, the original total area, and the volume-increasing rate respectively.

In the current study, the area was divided into 2000 identical rectangles using numerical integration to solve complex definite integrals. Since the numbers of cutting amounts were provided, the added value was almost equal to that of the original integral. In the proposed subject of the CAD model, for each value of pulling k (mm), the newly increased area and volume-increasing rate corresponding to 500 different tangential diameters were utilized for quantitative analysis.

### 2.11. Statistical Analysis

For this analysis, we accounted for repeated measurements on subjects using a linear mixed-effects model with a subject-specific random intercept. Volume and volume-increasing rate were treated as dependent variables, and area and length were treated as independent variables. The volume-increasing rate _i_ was calculated as [(Volume _at length = i_ − Volume _at length = 0_)/Volume _at length = 0_] × 100. The defective area was defined as a continuous variable and was used to designate three groups as follows: <125 cm^2^, 125–150 cm^2^, ≥150 cm^2^. The least squares mean (LS mean) was computed to estimate the marginal means over a balanced population. Statistical analysis was performed using SPSS version 22.0. A two-sided *p*-value <0.05 was considered as statistically significant.

## 3. Results

### 3.1. Patient Characteristics

From September 2013 to March 2014, 15 consecutive patients with TBI and stroke (ischemic and hemorrhagic) underwent DCs. Table 1 presents data on inflammation for DCs, including cause, intracranial pathology, and laterality.

### 3.2. 3D CAD Reconstruction to Simulate Brain Expansion Following DC

In clinical practice, neurosurgeons perform a wide DC to remove the skull bone and an outwardly additional space is, therefore, created, thereby protecting the brainstem against tissue compromise due to increased ICP. Firstly, we reconstructed CAD models with elevated contours to mimic the abovementioned phenomenon in creating additional extracranial space for brain expansion following DC. As shown in Figure 3 (case 3, as a representative patient), the newly reconstructed portion of the CAD model (an elevated contour from 2–50 mm) exhibited an outward bulging (labeled in blue), which corresponds to the clinical situation in patients with sustained injury-driven brain swelling.

**Figure 6:**
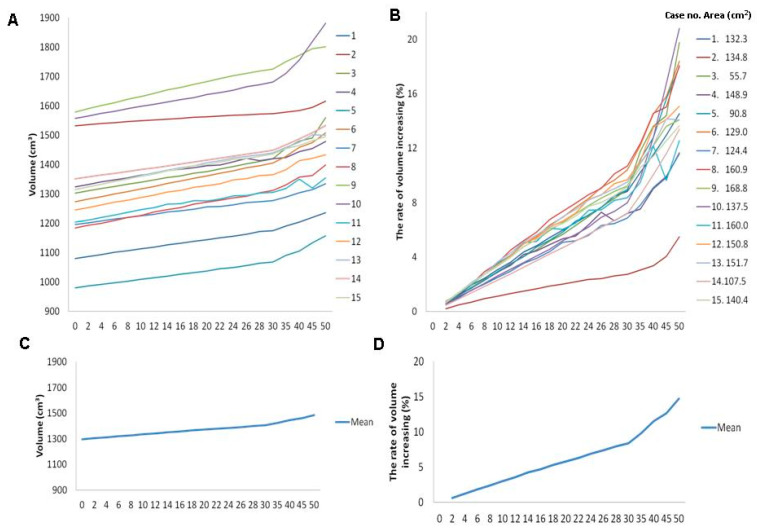
Individual profile of skull defect area (**right** panel of **B**) and plots for volume (**A**) and volume-increasing rate (**left** panel of **B**). Plots of mean profile for volume (**C**) and volume-increasing rate (**D**) in patients with contour elevation from 0 to 50 mm.

For all 15 subjects in the present study, volume and volume-increasing rate in CAD models at contour elevations of 2 to 50 mm are shown in Table 2 and Figure 6. The overall parameters of CAD models at contour elevations 0, 10, 20, 30, 40, and 50 mm were as follows: volume were 1296.4 ± 166.8, 1334.8 ± 168.5, 1370.7 ± 170.4, 1403.9 ± 172.2, 1444.3 ± 176.9, and 1486.3 ± 189.5 cm^3^, respectively; volume-increasing rates were 0%, 3.0% ± 0.7%, 5.8% ± 1.3%, 8.4% ± 1.9%, 11.5% ± 2.8%, and 14.7% ± 3.9%, respectively (as indicated in Table 2). The mean area of the skull defects (15 subjects) was 132.3 ± 29.7 cm^2^ (range of 55.7–168.8 cm^2^) (shown in the right panel, Figure 6B).

As demonstrated in Table 3, a significant increase was found in the volume and the volume-increasing rate, as the length of the outward contour increased. When compared to the regular CAD model, as contour elevation increased outward to 6 mm, statistical significance was achieved in the volume and the volume-increasing rate, and these parameters increased by 23.049 cm^3^ (*p* < 0.001) and 1.796% (*p* < 0.001), respectively. Eventually, as the contour elevation increased outward to 50 mm, the brain volume and the volume-increasing rate increased up to 189.840 cm^3^ and by 14.740%, respectively (*p* < 0.001). These results revealed a significant increase in the volume and the volume-increasing rate with an increase in the height of the reconstructed contour; this can be considered as a proxy for the phenomenon after DC procedure, where the scalp and the swollen brain tissue are observed to extend outward. The proposed CAD model with contour elevation was deemed to be sufficient to represent clinical brain swelling, and it was established for use in further quantitative analysis.

### 3.3. Increased Contour Elevation Height and Skull Defect Size Enhanced Volumetric Augmentation in the CAD Model

Following the observation in the CAD model that advanced contour elevation enabled space augmentation, we further asked whether this trend can be quantified as a clue for clinical consideration. Linear mixed-effects regression analysis was conducted to examine the parameters of the CAD model associated with volumetric expansion. As shown in Table 4, we found that the volume increased by 3.665 cm^3^ (standard error (SE): 0.067; *p*-value < 0.001) as the contour was reconstructed 1 mm outward. A 1 mm contour elevation was shown to promote the volume-increasing rate by 0.285% (SE: 0.005, *p*-value < 0.001). Moreover, for every 1 cm^2^ increase in the defect area of the skull, the volume-increasing rate was elevated by 0.034% (SE: 0.008; *p*-value < 0.001) (Table 3 and Table 4). These findings clearly disclosed that the volume-augmented effects can be effectively achieved either by elevating the scalp outward length or by removing the skull bone as much as possible during the DC procedure.

### 3.4. Interaction between the Elevation Height of the Contour and the Skull Defect Size and the Effect on Volumetric Augmentation in the CAD Model

As contour elevation and the area of the skull defect were quantitatively verified for association with volumetric augmentation, we then investigated the interaction trend between the two parameters. The 15 subjects were divided into three groups according to the defect area of the skull as follows: (i) Group 1, defect area <125 cm^2^; (ii) Group 2, defect area between 125–150 cm^2^; (iii) Group 3, defect area >150 cm^2^. As the contour was reconstructed 1 mm outward, the volume and the volume-increasing rate in Group 2 were found to be raised by 1.553 cm^3^ (*p* < 0.001) and 0.101% (*p* < 0.001), respectively, as compared to the respective parameters in Group 1. Moreover, a 1 mm elevation of contour reconstruction in Group 3 was shown to elevate the volume and the volume-increasing rate by 1.126 cm^3^ (*p* < 0.001) and 0.072% (*p* < 0.001), respectively, compared to those in Group 1 (Table 5). Thus, we postulated that elevating the scalp outward length can create greater volumetric augmentation under a larger defect area, which would provide the rationale that a wide DC (i.e., creating a greater skull defect) would be suggested during brain decompressive surgery.

### 3.5. Enhanced Effects of Elevation Length of the Contour in Volumetric Augmentation for a Larger Skull Defect Size

**Figure 7:**
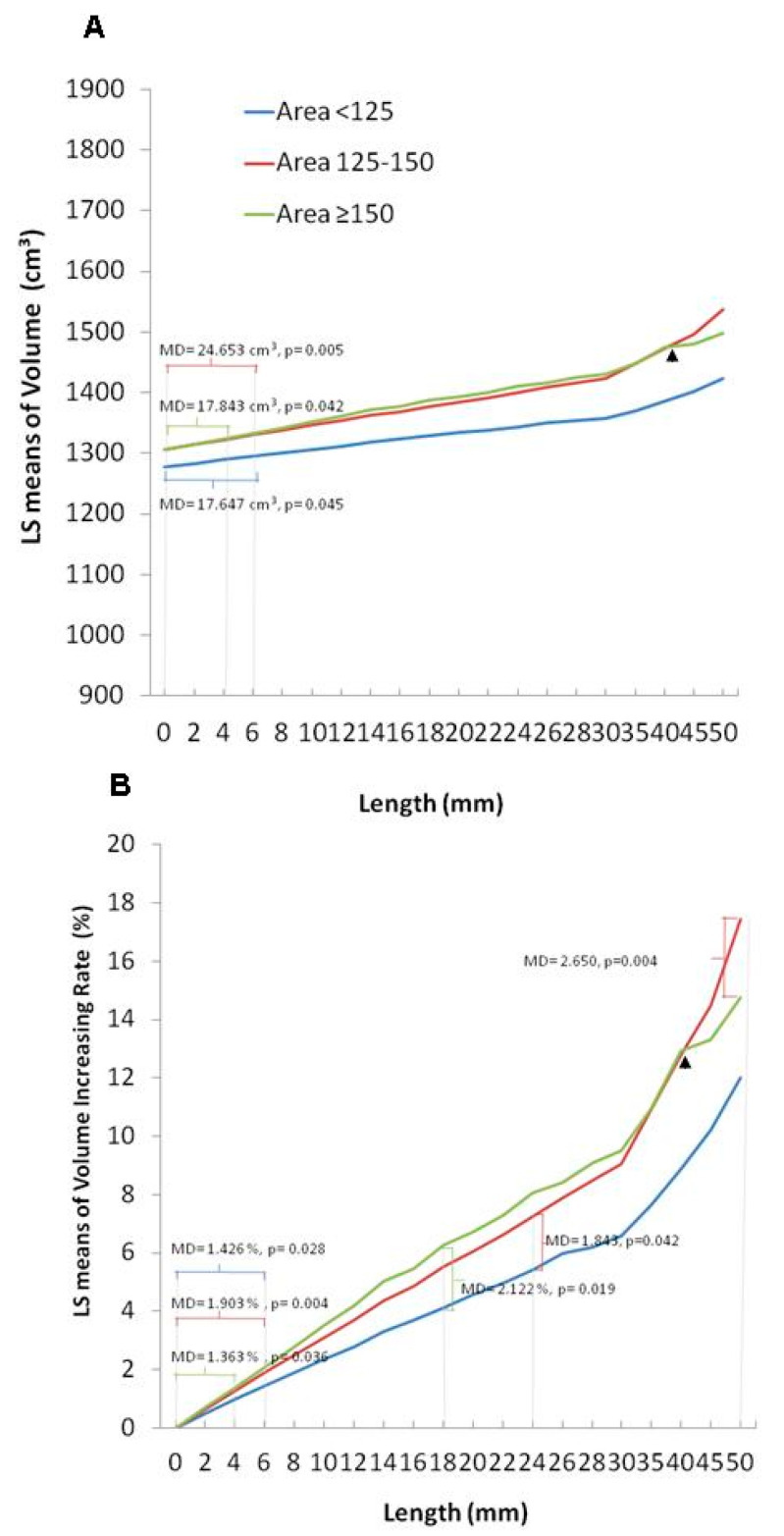
Estimated marginal means of volume (**A**) and volume-increasing rate (**B**) classified by area group in patients with contour elevation from 0 to 50 mm. LS: least squares; MD: mean difference.

Next, we sought to understand if the beneficial effects of contour elevation in volumetric augmentation were influenced by the size of the skull defects (in case the surgeons perform DC to an advanced extent). We investigated the effect of elevation length in enhancing volumetric augmentation by setting the skull defect size as a controlled factor. Thus, the 15 subjects were divided into Groups 1–3 according to the defect area of the skull, as described previously. In Groups 1 and 2, as the contour was reconstructed 6 mm outward; the differences were significant in the volume (mean difference = 17.647, *p* = 0.045; mean difference = 24.653, *p* = 0.005) and the volume-increasing rate (mean difference = 1.426, *p* = 0.028; mean difference = 1.9031, *p* = 0.004), respectively. In comparison, when the skull defect was extremely large in Group 3, a shorter length (4 mm) of contour elevation, compared to Group 1 and 2, contributed to significant differences in volume (mean difference = 17.843, *p* = 0.0426) and volume-increasing rate (mean difference = 1.363, *p* = 0.036). Specifically, we observed that, in each respective elevation length of the contour (2–40 mm), a greater extent of volume and volume-increasing rate was detected under advanced extents of skull defects (as indicated by respective higher levels of LS (least squares) mean changes, Group 3 > Group 2 > Group 1) (Table 6, Figure 7A, and the left panel of Figure 7B). These data support that the beneficial effects of contour elevation in volumetric expansion can be augmented with a larger skull defect size.

### 3.6. Abolished Efficacy of Elevation Length of the Contour in Volumetric Augmentation under Extremely Large Size of Skull Defect

We further sought to understand if an increase in the size of the skull defect results in increased volumetric expansion. As shown in Table 5, we observed that the volumetric augmentation achieved by contour elevation in Group 3 was inferior to that achieved in Group 2 (larger skull defects, but not to an extreme extent as in Group 3) (Group 2 vs. Group 3: 1.553 vs. 1.126 cm^3^ (raised volume); 0.101% vs. 0.072% (volume-increasing rate)), as the contour was reconstructed 1 mm outward. 

Furthermore, we set the elevation length as a control parameter and compared the difference between area groups in volumetric expansion. When Groups 2 and 1 were compared, statistical differences were not found in the volume-increasing rate until the reconstructed contour was elevated to 24 mm (mean difference = 1.843, *p* = 0.042). A shorter length of contour elevation was able to achieve effective volumetric augmentation in a wider range of defects in Group 3. At 18 mm of contour elevation, the differences in the volume-increasing rate between Groups 3 and 1 were significant (mean difference = 2.122, *p* = 0.019). Strikingly, when these two groups of large-scale skull defects were compared, it was difficult to establish an elevated contour to accomplish significant differences in volumetric augmentation. There was no difference in the volume-increasing rate between Groups 2 and 3 until the height of the contour reconstruction was raised to a very large number (>50 mm) (mean difference = 2.650, *p* = 0.004) (Table 7 and the right panel of Figure 7B). Note that with contour elevation values >40 mm, diminished levels of LS mean changes in the volume and the volume-increasing rate were observed in Group 3, compared to Group 2 (indicated by an arrow, Figure 7A,B).

These results obtained from the CAD models indicate that elevating the length of the contour alone is not effective enough to achieve volumetric expansion. The data showed that contour elevation combined with the creation of a larger skull defect is most effective. With large area defects, contour elevation can effectively and quickly achieve volumetric augmentation. However, if the area of the skull defect is larger than an optimum value, it is difficult to increase the volume even if the extent of the contour elongation is increased.

### 3.7. Plateau Status in Large Defect Area Presented in Ellipsoid Skull Model

Quantitative results from 3D CAD skull models with defect contour elevation showed that the volume-enhancing effect by contour elevation is not so effective under an extremely large skull defect. Next, the elliptical mathematical skull model was used to qualitatively verify the proposed plateau status under the circumstances of large defect area.

As shown in Figure 5A, we constructed three sets of ellipses: green, red, and blue ellipses. Respective Equation (4) are as follows:

(4) $$\text{Green ellipse:}\;\frac{{\left(x-h_{1}\right)}^{2}}{a_{1}^{2}}+\frac{y^{2}}{b_{1}^{2}}=1,\text{red ellipse:}\;\frac{{\left(x-h_{2}\right)}^{2}}{a_{2}^{2}}+\frac{y^{2}}{b_{2}^{2}}=1,\text{and blue ellipse:}\;\frac{{\left(x-h_{3}\right)}^{2}}{a_{3}^{2}}+\frac{y^{2}}{b_{3}^{2}}=1,$$

where *h*_1_, *h*_2_, and *h*_3_ represent the distance from point O to the midpoint of the respective ellipse on the *X*-axis, *b*_1_*b*_1_′, *b*_2_*b*_2_′, and *b*_3_*b*_3_′ represent the major axis along the *Y*-axis of the three sets of ellipses, and 2*b*_1_, 2*b*_2_, and 2*b*_3_ represent the respective length of the major axis. DC procedure-driven areas of the skull defect for *b*_1_*b*_1_′, *b*_3_*b*_3_′, and *b*_2_*b*_2_′ correspond to the smallest, largest, and second largest (close to the maximum skull defect *b*_3_*b*_3_*′*). Half of the elliptical arcs for the three sets of the skull defect (intercept points *b*_1_*b*_1_′, *b*_2_*b*_2_′, and *b*_3_*b*_3_*′*) are labeled in green, red, and blue, which refer to the curved contour formed after the scalp extends outward by height K in patients with different degrees of skull defects. Under this model of reconstructed contouring, the newly increased area is equal to half of the area under the contour of the proposed ellipse minus the overlapping area of the black ellipse.

We found that, if the length of the major axis is taken to be from a small to a large size (i.e., under different extents of DC-driven skull defects), the newly increased area will become larger. For example, the newly increased area under the red contour is larger than that under the green contour.

It is worth noting that, when the size of the DC-driven skull defect is larger than a certain size, e.g., greater than 2*b*_2_ (skull defect margin *b*_2_*b*_2_′), the newly added area under the reconstructed contour from the larger DC-driven skull defect will be gradually reduced. That is, the newly increased area under the red contour is larger than that of the blue contour (arrowed in Figure 5A) despite the length of 2*b*_3_ (blue) being larger than that of 2*b*_2_ (red). We postulate that, as the ellipse changes with the semi-minor axis and the semi-major axis, the curvature of the ellipse will also change. The abovementioned mathematical observations agree with the volumetric calculation of CAD model findings, i.e., that a larger size of a DC-driven skull defect is effective in volumetric augmentation, but the effect is attenuated when the skull defect is extremely large.

### 3.8. Quantitative Evaluation with Computerized Numerical Integration for Skull Defect Areas in Ellipsoid Skull Model

In numerical analysis, computational analysis for numerical integration with the rectangle method requires a given boundary value. For the computational analysis, let a = 65 mm and b = 75 mm (assuming the general skull size).

Firstly, let K = 10 mm. We found that, as the tangent diameter increases, the corresponding newly increased area and the volume-increasing rate also increase. However, when the tangent diameter exceeds approximately 143.55 mm, the newly increased area and the volume-increasing rate begin to decrease (Figure S1A,B, Supplementary Materials, respectively). Specifically, as shown in Figure S1A,B (Supplementary Materials), when the tangential diameter is about 143.55, the newly increased area and volume-increasing rate reach about 1461.63 and 9.55%, respectively. In comparison, when the tangential diameter almost reaches the maximum at 150, the newly increased area and the volume-increasing rate drop to about 1179.53 and 7.7%, respectively (as shown in Texts S1 and S2, Supplementary Materials).

Using the same algorithm as used for the computational analysis, we verified the trend in situations where K = 20, 30, 40, and 50 mm. The corresponding plateau values of the tangential diameter were 145.54, 146.72, 147.44, and 147.92 respectively (as indicated in Figures S1–S5, Supplementary Materials). The corresponding plateau values of the newly increased area and the volume-increasing rate were as follows: 2598.53 mm^3^, 16.96% (K = 20) (Figure S2A,B, Supplementary Materials); 3747.17 mm^3^, 24.46% (K = 30) (Figure S3A,B, Supplementary Materials); 4902.89 mm^3^, 32.003 (K = 40) (Figure S4A,B, Supplementary Materials); 6063.32 mm^3^, 39.58% (K = 50) (Figure S5A,B, Supplementary Materials). In comparison, when the tangential diameter almost reached the maximum at 150, the newly increased area and the volume-increasing rate decreased to 2358.28 mm^3^, 15.39% (K = 20) (Texts S3 and S4, Supplementary Materials), 3536.91 mm^3^, 23.09% (K = 30) (Texts S5 and S6, Supplementary Materials), 4715.50 mm^3^, 30.78% (K = 40) (Texts S7 and S8, Supplementary Materials), and 5894.06 mm^3^, 38.47% (K = 50) (Texts S9 and S10, Supplementary Materials), respectively. The following trend was consistently observed: when the tangent diameter was larger than the respective plateau value, the newly increased area and the volume-increasing rate declined. Specifically, we found that, when the value of k increased, the tangent diameter corresponding to the maximum value of the newly increased area and the volume-increasing rate also increased to a level approximating that of 2b.

### 3.9. Mathematical Algorithm for Simulating Contour Elevation Using an Ellipsoid Skull Model in Potential Clinical Practice

Our data from the CAD models revealed interactions between the elevation length of the contour and the skull defect size in volumetric augmentation. Lastly, we attempted to identify suitable parameters. As shown in a clinical report of the human scalp, wound diameter greater than 41 mm was found to be too large for wound closure [13]. We, therefore, interpret that the scalp can be extended up to 41 mm in width. Figure 5C shows an ellipsoid model as a proxy of the skull. In this original ellipse, we assume that the arc length of the defect margin is X, the length of the major axis is a, and the length of the minor axis is b. Additionally, if we assume that the maximum of the contour elevation height is K, the arc can be extended to as long as X + 4.1. In this new ellipse, the length of the major axis is a, and the length of the minor axis is b + K.

According to the formula for the arc length of an ellipse, i.e., 2πb + 4(a − b), we can obtain the following:

Length of new arc/Length of original arc
= (X + 4.1)/X
= [2π(b + K) + 4(a − b − k)]/[2πb + 4(a − b)].

K is, thus, obtained and is equal to [16.4a + (8.2π − 16.4)b/X(2π − 4).

Let π be 3.14.

K is calculated to be (16.4a + 9.348b)/2.28X.

Again, assuming that a = 65 mm and b = 75 mm, as a proxy of the general skull size, X can be calculated as 224.1 mm, and K is obtained as 3.596 mm. 

## 4. Discussion

Under the circumstances of brain insults, primary injury immediately impairs osmolite transport, enhances oxidative stress, and triggers an inflammatory cascade, thereby leading to devastating secondary injuries and consequent brain swelling [14,15]. Within the limited cranial vaults, such injury-triggered brain edema causes an increase in the ICP, a decrease in the cerebral blood flow, and an attenuation of the oxygen supply to the brain, thereby resulting in amplified brain swelling. As this vicious pathological cycle continues, sustained brain swelling may eventually lead to brainstem compromise and cause potentially life-threatening complications [16]. The DC procedure is indicated in patients suffering from advanced brain swelling, such as TBI-induced diffuse brain edema [17]. However, in some situations, such as spontaneous intracerebral hemorrhage, the brain swelling is not as severe as that caused by trauma. At such minor circumstances, for long-term prognosis, the effect of DC treatment may be worsened by bone replacement after hematoma evacuation, on account of sequelae caused by DC [18].

The following methods have been addressed in current research to study the therapeutic effect of DC: (1) finite element (FE) model in experiment [19] and 3D editing FE model from magnetic resonance imaging [20] to investigate mechanical strain or brain biomechanical properties; (2) 3D printing artificial skull model from CT for skull defect-related quantitation [21]; (3) CT-based quantitative analysis in patients for skull defect [22] or contour elevation height-associated quantitation [23,24]; (4) hypothetic model of cerebral hemispheres with mathematical algorithm for quantitative assessment of transcalvarial brain herniation volume from skull effect [25]. Our data showed that the volume and volume-increasing rate were statistically significant when contour elevation height exceeded 6 mm. In correspondence with our results, CT-based quantitative analysis in 56 TBI patients demonstrated a 5 mm cutoff value [23,26] (i.e., elevating contour); this value achieved sufficient space for brain swelling without causing difficulties in scalp adaptation [27,28].

The analysis of craniectomy size has been addressed in a number of studies to assess the postoperative outcomes in patients following DC. A correlation between craniectomy size and the potential expanded volume postoperatively has been reported in a 3D printing skull model [21] and 3D editing FE model [20]. A minimum diameter of 8.3 [21] or 12 cm [29] has been reported to lower increased ICP. Moreover, a CT-based research of 30 TBI patients indicated that an effective control of ICP can be achieved with a ratio (defective circumference/skull circumference) greater than 65% [22]. Craniectomy size has been shown to correlate with decreased mortality [29]. However, for long-term follow-up, eventual outcomes were either not significantly [30] or inconclusively [29] related to the craniectomy size.

The quantification of brain expansion volume can also be performed on a hypothetic mathematical model of defective skulls. A similar ellipsoid model to our study used CAD data for conformation, whereby volume creation could then be approximately calculated as 1/2ACΔh [25] (A,C: base diameters; ∆h: spherical cap height difference). In this model, assuming a radius 80 mm for half sphere, the maximum volume increase can subsequently be calculated under the status of a = 70.5 mm and Δh = 283 mm. This calculated result was consistent with the trend of our research. The maximal volume expansion was not generated in the largest diameter (8 cm) of skull defect. However, a proposed boundary value needed to be given, such as 80 mm radius length in this report and 75 mm in our ellipsoid skull model, which possibly contributes a minor simulated effect compared to the CAD model or neuroimage-based 3D model that can indicate trends for brain microdynamics in quantitative analysis. To compare, CAD-based quantitative assessments aid to gain a better understanding of the relationship among these pivotal parameters related to DC, i.e., outward brain expansion, skull defect area, scalp-elevating length, and brainstem dynamics (better visualization for brainstem in CAD), such as the case in our investigation [31].

To obtain a closer simulation, 3D CAD models from patients following DC were constructed to quantitatively assess and further mathematically verify the effect of the size of the skull defect and the elevation of the length of the contour on volumetric augmentation. Our data showed that removing the skull bone as much as possible was beneficial in terms of volume augmentation (0.034% elevation (volume-increasing rate) for every 1 cm^2^ increase (defect area of the skull)). Moreover, an impaired volume-augmentation effect was revealed when the skull defect was extremely large; this aspect has not been addressed in previous qualitative studies. Herein, we recommend that the maximum boundary of the skull defect should be within the following vital structures: the superior sagittal sinus superomedially, the transverse sinus posteroinferiorly, the frontal sinus anteroinferiorly, and the frontozygomatic junction lateral inferiorly. Our suggestions are in agreement with previous publications [32] (craniectomy margins: 4 cm above the midpupil anteriorly, the parieto-occipital point posteriorly, larger than 2.5 cm from the midline superiorly [33], and 1 cm above the zygomatic arch inferiorly), but with a modification (extended posteriorly to achieve maximal craniectomy limit). As suggested by our data, an extremely large skull defect exceeding the abovementioned boundary is not desirable, because of issues such as potentially reduced volume expansion capability and an increased risk of bleeding and complications. 

As of now, practical surgical guidance and quantitative suggestions are not available to help prevent intraoperative brain extrusion and to achieve optimal extent of DC, respectively. Because of the lack of objective evaluation of brain expansion after DC, widely adopted surgical guidelines for DC procedures do not exist. Such guidelines are needed for the standardized clinical assessment of patients who present with profound brain swelling with emergent DC surgeries. Various surgical strategies including discontinuous dural fenestration [34] and duraplasty desertion covered with Surgicel [35] have been suggested to shorten surgical time and, therefore, reduce the risk for operative brain extrusion. We believe that the current quantitative research provides a clinical reference to formulate individualized remedies for patients undergoing DC surgery. 

This study was subjected to a number of limitations. Although the data obtained were statistically significant, the study included a relatively small number of CAD subjects and did not include CAD models with bilateral skull defects. Regarding bilateral skull defects, we recommend the following procedure: fill the skull defect on one side with a symmetrical concept as part of an elliptical arc; then, symmetrically apply mirror-reflections of the proposed contour to the skull defect of the opposite side. The subsequent steps are similar to those applied for unilateral defects. Second, CAD models included in the current study represent the size and head shape of the oriental population. If these models are to be used globally, information pertaining to Western populations needs to be added to recalculate and reanalyze the data. Lastly, we utilized CAD modeling to simulate brain swelling, which represents, for the majority, an ideal status for a swollen brain. However, significant features influencing brain expansion such as cerebral compliance and brain tissue stiffness [36] cannot be completely simulated in the modeling method. Thus, the detailed mechanisms involved in brain swelling and advanced CAD designs need further investigation.

## 5. Conclusions

Using a CAD model including enough algorithms for simulating the outward extension of the scalp (50 mm) and various areas of skull defects, our data showed the following: (1) outward contour (scalp) elevation and removal of a larger portion of the skull bone enhanced volume-augmentation effects; (2) volume-augmentation effects of contour elevation can be effectively achieved in case of large area defects; (3) efficiency of volumetric augmentation by contour elevation can be reduced when the skull defect areas are extremely large. The contour elevation height can be obtained using elliptical equations, such as the following: (16.4a + 9.348b)/2.28X (a: distance from the center point of the skull to the anterior part of the skull; b: distance centrally to laterally; X: arc length of the defect margin). We recommend skull defect areas with a maximal edge within the following critical structures: superior sagittal sinus superomedially, transverse sinus posteroinferiorly, frontal sinus anteroinferiorly, and frontozygomatic junction lateral inferiorly.

## Figures and Tables

**Table 1:** Demographic data of 15 subjects.

Patient	Cause for DC	TBI Mechanism	Hematoma/Infarction Location	DC
1	TBI	Motor vehicle accident	Acute SDH, left F-T	Uni + HR
2	Hemorrhagic stroke	-	ICH, left BG	Uni + HR
3	Left MCA Infarction	-	Left MCA territory	Uni
4	TBI	Fall	SDH, right F-T	Uni + HR
5	TBI	Impact	Acute EDH,SDH, left F-T	Uni + HR
6	TBI	Motor vehicle accident	Acute SDH, right F-T; acute ICH, right F	Uni + HR
7	Left MCA Infarction	-	left MCA territory	Uni
8	TBI	Fall	Acute SDH, left F-T	Uni + HR
9	TBI	Motor vehicle accident	Acute SDH, right T	Uni + HR
10	TBI	Motor vehicle accident	Acute SDH, right F-T	Uni + HR
11	Right MCA Infarction	-	Right MCA territory	Uni
12	TBI	Fall	Acute EDH,SDH, ICH, right T-P	Uni + HR
13	Hemorrhagic stroke	-	ICH, right BG	Uni + HR
14	TBI	Fall	Acute SDH, left T-P	Uni + HR
15	TBI	Motor vehicle accident	Acute SDH, right F-T, ICH, right F	Uni + HR

M, male; F, female; TBI, traumatic brain injury; EDH, epidural hematoma; SDH, subdural hematoma; ICH, intracerebral hematoma; BG, basal ganglion; F, frontal; T, temporal; P, parietal; DC, decompressive craniectomy; Uni + HR, unilateral craniectomy + removal of hematoma; Uni, unilateral DC without removal of hematoma.

**Table 2:** Parameter description of 15 CAD models.

Length of Contour Elevation (mm)	Volume (cm^3^)					Volume-Increasing Rate (%)				
	Mean	SD	Median	Min	Max	Mean	SD	Median	Min	Max
**0**	1296.4	166.8	1303.0	980.8	1579.7					
2	1304.1	167.1	1311.0	987.0	1590.5	0.6	0.1	0.6	0.2	0.8
4	1311.9	167.8	1318.6	992.4	1602.1	1.2	0.3	1.3	0.5	1.4
6	1319.5	167.7	1326.3	997.9	1611.0	1.8	0.4	2.0	0.7	2.1
8	1327.1	168.4	1333.9	1003.5	1622.9	2.4	0.5	2.5	1.0	2.9
10	1334.8	168.5	1340.7	1009.8	1632.5	3.0	0.7	3.1	1.1	3.7
12	1342.1	168.8	1348.9	1015.3	1642.8	3.6	0.8	3.6	1.3	4.5
14	1350.7	169.1	1356.1	1020.3	1655.0	4.2	1.0	4.4	1.5	5.2
16	1356.4	169.4	1362.3	1027.4	1663.0	4.7	1.1	4.8	1.7	5.8
18	1364.5	169.7	1370.4	1032.3	1673.7	5.3	1.2	5.4	1.9	6.8
20	1370.7	170.4	1377.0	1038.0	1683.1	5.8	1.3	6.0	2.0	7.4
22	1377.1	170.8	1385.5	1046.1	1693.4	6.3	1.4	6.5	2.2	8.0
24	1385.1	171.3	1394.1	1050.0	1702.7	6.9	1.6	7.1	2.4	8.6
26	1391.7	172.1	1403.0	1056.0	1711.5	7.4	1.6	7.7	2.4	9.1
28	1398.0	171.3	1411.5	1064.2	1719.0	7.9	1.8	8.5	2.6	10.1
30	1403.9	172.2	1420.0	1068.4	1725.3	8.4	1.9	8.9	2.7	10.7
35	1422.5	174.1	1428.9	1090.4	1751.7	9.8	2.4	10.6	3.1	12.3
40	1444.3	176.9	1458.5	1106.3	1772.0	11.5	2.8	12.2	3.4	14.6
45	1459.6	185.1	1474.7	1134.1	1818.6	12.7	3.3	13.6	4.1	16.7
50	1486.3	189.5	1495.4	1157.2	1881.9	14.7	3.9	14.1	5.5	20.8

**Table 3:** Parameter estimation of 15 CAD models: length of contour elevation as categorical data.

Effects	Dependent Variable			
	Volume (cm^3^)		Volume-Increasing Rate (%)	
	Estimate (SE)	*p*-Value	Estimate (SE)	*p*-Value
Intercept	1237.580 (215.630)	<0.0001	−4.443 (1.106)	0.002
Area *	0.445 (1.593)	0.785	0.034 (0.008)	0.001
Length ^#^				
0	0		0	
2	7.681 (6.053)	0.206	0.599 (0.429)	0.164
4	15.465 (6.053)	0.011	1.202 (0.429)	0.005
6	23.049 (6.053)	<0.001	1.796 (0.429)	<0.001
8	30.643 (6.053)	<0.001	2.385 (0.429)	<0.001
10	38.390 (6.053)	<0.001	2.991 (0.429)	<0.001
12	45.697 (6.053)	<0.001	3.560 (0.429)	<0.001
14	54.247 (6.053)	<0.001	4.227 (0.429)	<0.001
16	59.982 (6.053)	<0.001	4.675 (0.429)	<0.001
18	68.036 (6.053)	<0.001	5.304 (0.429)	<0.001
20	74.244 (6.053)	<0.001	5.785 (0.429)	<0.001
22	80.676 (6.053)	<0.001	6.288 (0.429)	<0.001
24	88.721 (6.053)	<0.001	6.913 (0.429)	<0.001
26	95.250 (6.053)	<0.001	7.419 (0.429)	<0.001
28	101.540 (6.053)	<0.001	7.922 (0.429)	<0.001
30	107.490 (6.053)	<0.001	8.381 (0.429)	<0.001
35	126.110 (6.053)	<0.001	9.833 (0.429)	<0.001
40	147.930 (6.053)	<0.001	11.528 (0.429)	<0.001
45	163.170 (6.053)	<0.001	12.671 (0.429)	<0.001
50	189.840 (6.053)	<0.001	14.740 (0.429)	<0.001

Standard error: SE; ^#^ length of contour elevation (length) (mm); * areas of skull defects (area) (cm^2^).

**Table 4:** Parameter estimation for volume and volume-increasing rate of 15 CAD models based on length of contour elevation and areas of skull defects.

Effects	Dependent Variable			
	Volume (cm^3^)		Volume-Increasing Rate (%)	
	Estimate (SE)	*p*-Value	Estimate (SE)	*p*-Value
Intercept	1238.350 (215.600)	<0.001	−4.376 (1.070)	0.001
Area of skull defects (cm^2^)	0.445 (1.593)	0.78	0.034 (0.008)	<0.001
Length of contour elevation (mm)	3.665 (0.067)	<0.001	0.285 (0.005)	<0.001

**Table 5:** Interaction between elevation height of contour and skull defect size for volume and volume-increasing rate of 15 CAD models.

Effects	Dependent Variable			
	Volume (cm^3^)		Volume-Increasing Rate (%)	
	Estimate (SE)	*p*-Value	Estimate (SE)	*p*-Value
Intercept	1277.82 (81.623)	<0.001	0.014 (0.477)	0.977
Group *				
1	0		0	
2	23.63 (115.43)	0.84	−0.311 (0.674)	0.65
3	34.448 (115.43)	0.77	0.472 (0.674)	0.50
Length ^#^	2.772 (0.094)	<0.001	0.228 (0.007)	<0.001
Group × Length				
1	0		0	
2	1.553 (0.134)	<0.001	0.101 (0.01)	<0.001
3	1.126 (0.134)	<0.001	0.072 (0.01)	<0.001

* Skull defect size: Group 1: area <125 cm^2^; group 2: 125 cm^2^ ≤ area <150 cm^2^; group 3: area ≥150 cm^2^; ^#^ length of contour elevation (length) (mm).

**Table 6:** Comparison of the effects of contour elevation on volume expansion among Groups 1–3 *.

Length of Contour Elevation (mm)	Group 1				Group 2				Group 3			
	Volume (cm^3^)		Volume-Increasing Rate (%)		Volume (cm^3^)		Volume-Increasing Rate (%)		Volume (cm^3^)		Volume-Increasing Rate (%)	
	LS Mean Change	*p*-Value	LS Mean Change	*p*-Value	LS Mean Change	*p*-Value	LS Mean Change	*p*-Value	LS Mean Change	*p*-Value	LS Mean Change	*p*-Value
2	5.917	0.499	0.480	0.458	8.176	0.350	0.630	0.331	8.949	0.307	0.685	0.290
4	12.104	0.167	0.975	0.132	16.449	0.061	1.267	0.051	17.843	0.042	1.363	0.036
6	17.647	0.045	1.426	0.028	24.653	0.005	1.903	0.004	26.848	0.002	2.058	0.002
8	23.389	0.008	1.887	0.004	32.239	0.000	2.483	0.000	36.302	<0.0001	2.784	<0.0001
10	29.281	0.001	2.368	0.000	40.153	<0.0001	3.094	<0.0001	45.734	<0.0001	3.510	<0.0001
12	34.527	0.000	2.794	<0.0001	47.812	<0.0001	3.682	<0.0001	54.752	<0.0001	4.205	<0.0001
14	40.908	<0.0001	3.306	<0.0001	56.395	<0.0001	4.350	<0.0001	65.438	<0.0001	5.025	<0.0001
16	45.727	<0.0001	3.706	<0.0001	62.963	<0.0001	4.852	<0.0001	71.256	<0.0001	5.466	<0.0001
18	51.016	<0.0001	4.132	<0.0001	71.671	<0.0001	5.524	<0.0001	81.422	<0.0001	6.254	<0.0001
20	56.612	<0.0001	4.593	<0.0001	78.560	<0.0001	6.049	<0.0001	87.559	<0.0001	6.712	<0.0001
22	60.898	<0.0001	4.954	<0.0001	86.125	<0.0001	6.632	<0.0001	95.004	<0.0001	7.278	<0.0001
24	66.843	<0.0001	5.423	<0.0001	94.369	<0.0001	7.266	<0.0001	104.950	<0.0001	8.050	<0.0001
26	73.600	<0.0001	5.975	<0.0001	102.380	<0.0001	7.874	<0.0001	109.770	<0.0001	8.407	<0.0001
28	76.000	<0.0001	6.194	<0.0001	110.010	<0.0001	8.472	<0.0001	118.600	<0.0001	9.101	<0.0001
30	80.878	<0.0001	6.586	<0.0001	117.870	<0.0001	9.064	<0.0001	123.740	<0.0001	9.493	<0.0001
35	93.144	<0.0001	7.632	<0.0001	142.310	<0.0001	10.922	<0.0001	142.860	<0.0001	10.947	<0.0001
40	108.350	<0.0001	8.873	<0.0001	167.300	<0.0001	12.777	<0.0001	168.160	<0.0001	12.933	<0.0001
45	124.330	<0.0001	10.214	<0.0001	190.940	<0.0001	14.490	<0.0001	174.230	<0.0001	13.310	<0.0001
50	146.920	<0.0001	12.021	<0.0001	230.440	<0.0001	17.425	<0.0001	192.160	<0.0001	14.775	<0.0001

* Skull defect size: Group 1: area <125 cm^2^; group 2: 125 cm^2^ ≤ area <150 cm^2^; group 3: area ≥150 cm^2^. Difference was assessed using a mixed model for repeated measurement analysis. LS, least squares.

**Table 7:** Comparison of the effects of contour elevation on volume expansion between Groups *.

Length of Contour Elevation (mm)	Group 2 vs. 1				Group 2 vs. 3			Group 3 vs. 1			
	Volume (cm^3^)		Volume-Increasing Rate (%)		Volume (cm^3^)		Volume-Increasing Rate (%)	Volume (cm^3^)		Volume-Increasing Rate (%)	
	LS Mean Difference	*p*-Value	LS Mean Difference	*p*-Value	LS Mean Difference	*p*-Value	LS Mean Difference	LS Mean Difference	*p*-Value	LS Mean Difference	*p*-Value
0	28.826	0.804	0.000	1.000	0.161	0.999	0.000	28.665	0.805	0.000	1.000
2	31.085	0.789	0.150	0.868	−0.612	0.996	−0.055	31.696	0.784	0.205	0.820
4	33.170	0.775	0.292	0.746	−1.233	0.992	−0.096	34.403	0.767	0.387	0.668
6	35.831	0.757	0.477	0.597	−2.035	0.986	−0.155	37.866	0.744	0.632	0.483
8	37.675	0.745	0.596	0.508	−3.902	0.973	−0.301	41.577	0.720	0.897	0.320
10	39.697	0.732	0.727	0.420	−5.420	0.963	−0.416	45.118	0.697	1.142	0.206
12	42.110	0.716	0.889	0.325	−6.779	0.953	−0.523	48.890	0.673	1.411	0.118
14	44.313	0.702	1.044	0.247	−8.882	0.939	−0.676	53.195	0.646	1.720	0.057
16	46.062	0.691	1.146	0.204	−8.132	0.944	−0.613	54.194	0.640	1.760	0.052
18	49.481	0.669	1.392	0.123	−9.590	0.934	−0.730	59.071	0.610	2.122	0.019
20	50.774	0.661	1.456	0.107	−8.838	0.939	−0.663	59.612	0.607	2.120	0.019
22	54.053	0.641	1.679	0.064	−8.717	0.940	−0.646	62.770	0.588	2.324	0.010
24	56.352	0.627	1.843	0.042	−10.421	0.928	−0.784	66.773	0.565	2.627	0.004
26	57.609	0.619	1.899	0.036	−7.223	0.950	−0.533	64.831	0.576	2.432	0.007
28	62.838	0.588	2.278	0.012	−8.430	0.942	−0.629	71.268	0.539	2.907	0.001
30	65.815	0.570	2.478	0.006	−5.710	0.961	−0.429	71.525	0.537	2.907	0.001
35	77.995	0.501	3.290	0.000	−0.390	0.997	−0.025	78.385	0.499	3.315	0.000
40	87.776	0.449	3.905	<0.0001	−0.700	0.995	−0.156	88.476	0.445	4.061	<0.0001
45	95.435	0.410	4.275	<0.0001	16.874	0.884	1.180	78.561	0.498	3.096	0.001
50	112.350	0.333	5.404	<0.0001	38.437	0.740	2.650	73.912	0.524	2.753	0.003

* Skull defect size: Group 1: area <125 cm^2^; group 2: 125 cm^2^ ≤ area <150 cm^2^; group 3: area ≥150 cm^2^. Difference was assessed using a mixed model for repeated measurement analysis. LS, least squares.

## Data Availability

The data used to support the findings of this study are included within the article.

## Supplementary Materials

The following are available online at https://www.mdpi.com/article/10.3390/brainsci11040428/s1. Supplementary Figure S1. Plots of numerical integration for volume (A) and volume-increasing rate (B) in the proposed ellipse model with various tangent diameters (a = 65, b = 75, K = 10 mm), Supplementary Figure S2. Plots of numerical integration for volume (A) and volume-increasing rate (B) in the proposed ellipse model with various tangent diameters (a = 65, b = 75, K = 20 mm), Supplementary Figure S3. Plots of numerical integration for volume (A) and volume-increasing rate (B) in the proposed ellipse model with various tangent diameters (a = 65, b = 75, K = 30 mm), Supplementary Figure S4. Plots of numerical integration for volume (A) and volume-increasing rate (B) in the proposed ellipse model with various tangent diameters (a = 65, b = 75, K = 40 mm), Supplementary Figure S5. Plots of numerical integration for volume (A) and volume-increasing rate (B) in the proposed ellipse model with various tangent diameters (a = 65, b = 75, K = 50 mm), Supplementary Text S1 Results for volume in the proposed ellipse model with various tangent diameters (a = 65, b = 75, K = 10 mm), Supplementary Text S2 Results for volume-increasing rate in the proposed ellipse model with various tangent diameters (a = 65, b = 75, K = 10 mm), Supplementary Text S3 Results for volume in the proposed ellipse model with various tangent diameters (a = 65, b = 75, K = 20 mm), Supplementary Text S4 Results for volume-increasing rate in the proposed ellipse model with various tangent diameters (a = 65, b = 75, K = 20 mm), Supplementary Text S5 Results for volume in the proposed ellipse model with various tangent diameters (a = 65, b = 75, K = 30 mm), Supplementary Text S6 Results for volume-increasing rate in the proposed ellipse model with various tangent diameters (a = 65, b = 75, K = 30 mm), Supplementary Text S7 Results for volume in the proposed ellipse model with various tangent diameters (a = 65, b = 75, K = 40 mm), Supplementary Text S8 Results for volume-increasing rate in the proposed ellipse model with various tangent diameters (a = 65, b = 75, K = 40 mm), Supplementary Text S9 Results for volume in the proposed ellipse model with various tangent diameters (a = 65, b = 75, K = 50 mm), Supplementary Text S10 Results for volume-increasing rate in the proposed ellipse model with various tangent diameters (a = 65, b = 75, K = 50 mm).
