# Supplementary material for: Simulating Expansion of the Intracranial Space to Accommodate Brain Swelling after Decompressive Craniectomy: Volumetric Quantification in a 3D CAD Skull Model with Contour Elevation
Source: Brain Sci. 2021 Mar 27;11(4):428. doi: 10.3390/brainsci11040428 (PMC8067154; doi:10.3390/brainsci11040428)
Supplement: Supplementary file 1 [file brainsci-11-00428-s001.pdf]

Supplementary figure legends

Supplementary Fig.S1. Plots of numerical integration for volume (A) and volume-increasing rate (B) in the proposed ellipse model with various tangent diameters (a=65, b=75, K=10 mm).

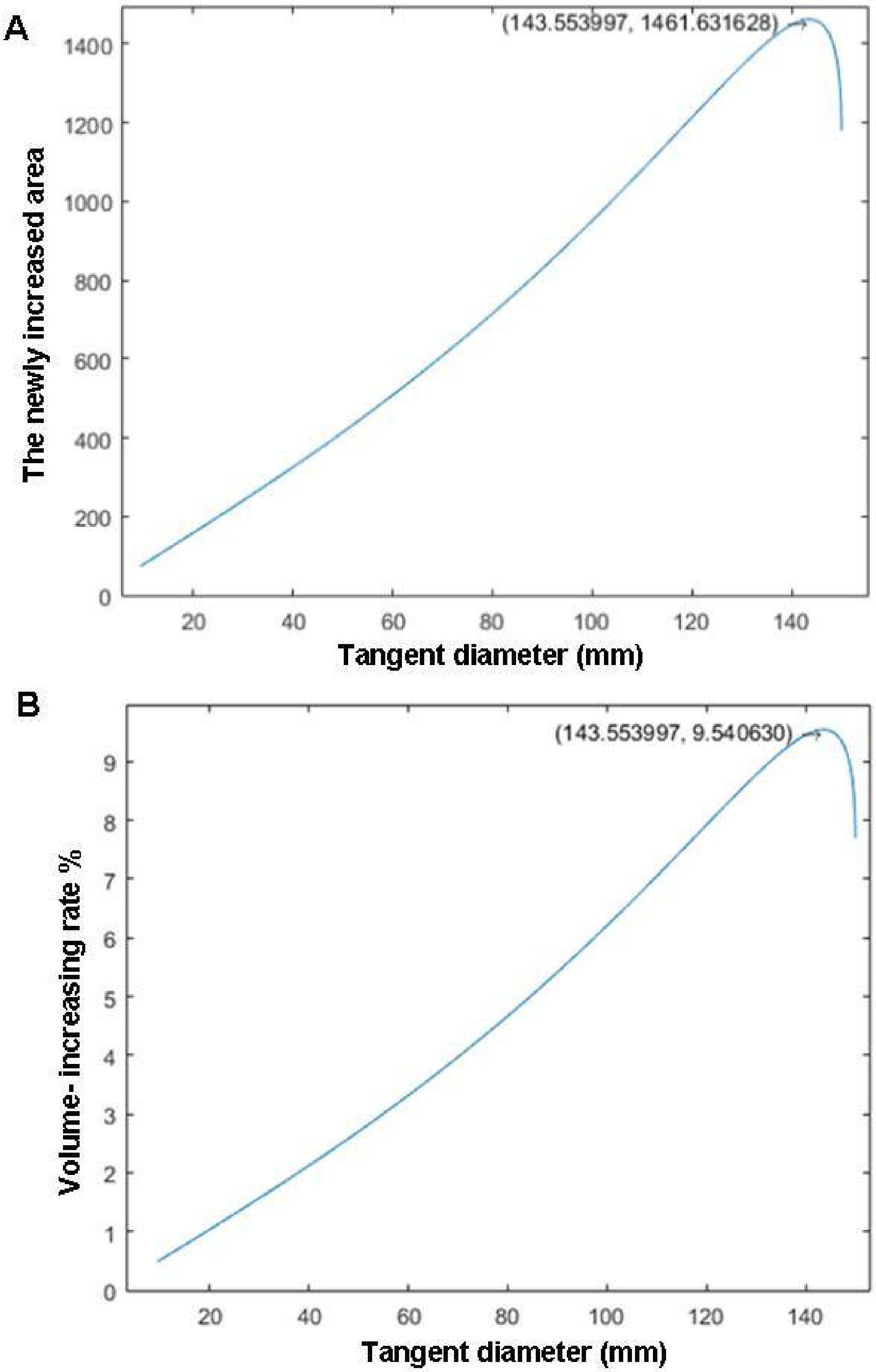
A
(143.553997, 1461.631628)
The newly increased area
0
200
400
600
800
1000
1200
1400
20
40
60
80
100
120
140
Tangent diameter (mm)
B
(143.553997, 9.540630)
Volume- increasing rate %
0
1
2
3
4
5
6
7
8
9
20
40
60
80
100
120
140
Tangent diameter (mm)

Supplementary Fig.S2. Plots of numerical integration for volume (A) and volume-increasing rate (B) in the proposed ellipse model with various tangent diameters (a=65, b=75, K=20mm).

A

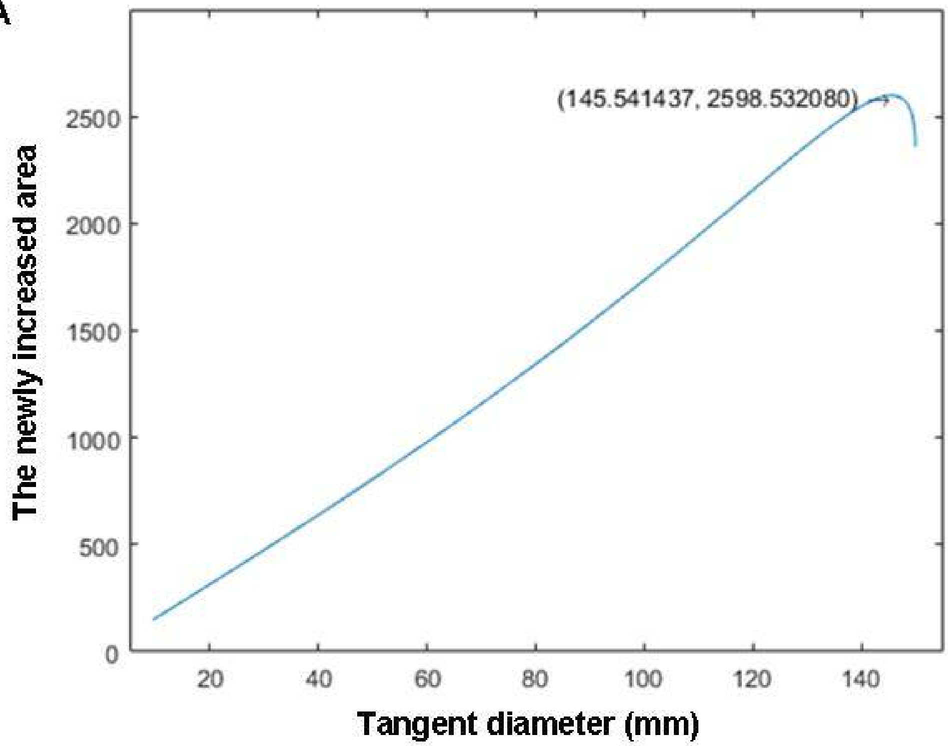

(145.541437, 2598.532080)
The newly increased area
Tangent diameter (mm)

B

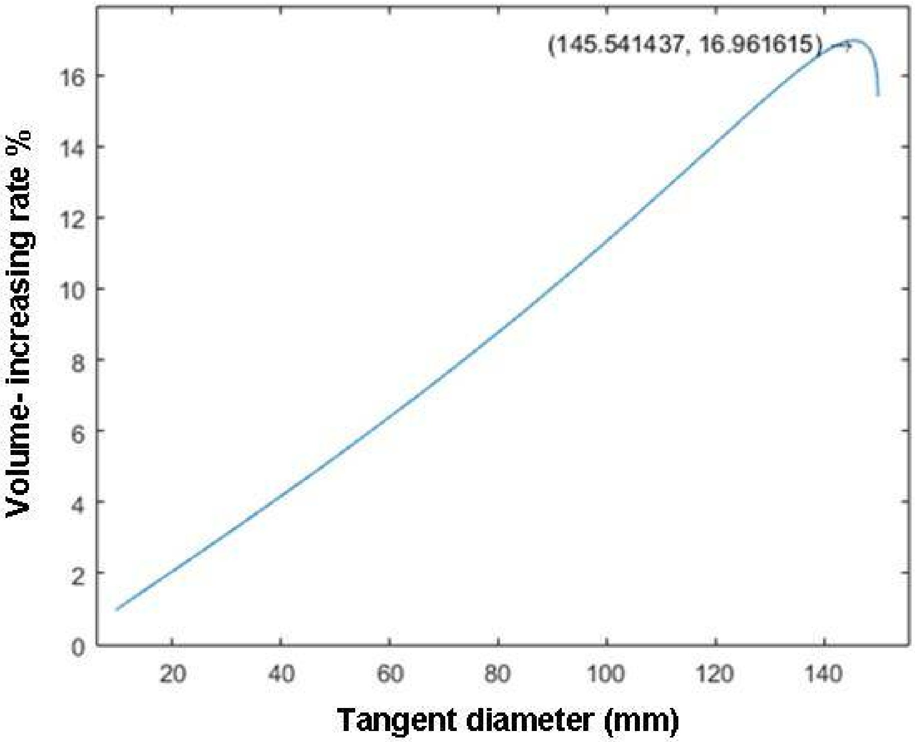

(145.541437, 16.961615)
Volume- increasing rate %
Tangent diameter (mm)

Supplementary Fig.S3. Plots of numerical integration for volume (A) and volume-increasing rate (B) in the proposed ellipse model with various tangent diameters (a=65, b=75, K=30mm).

A

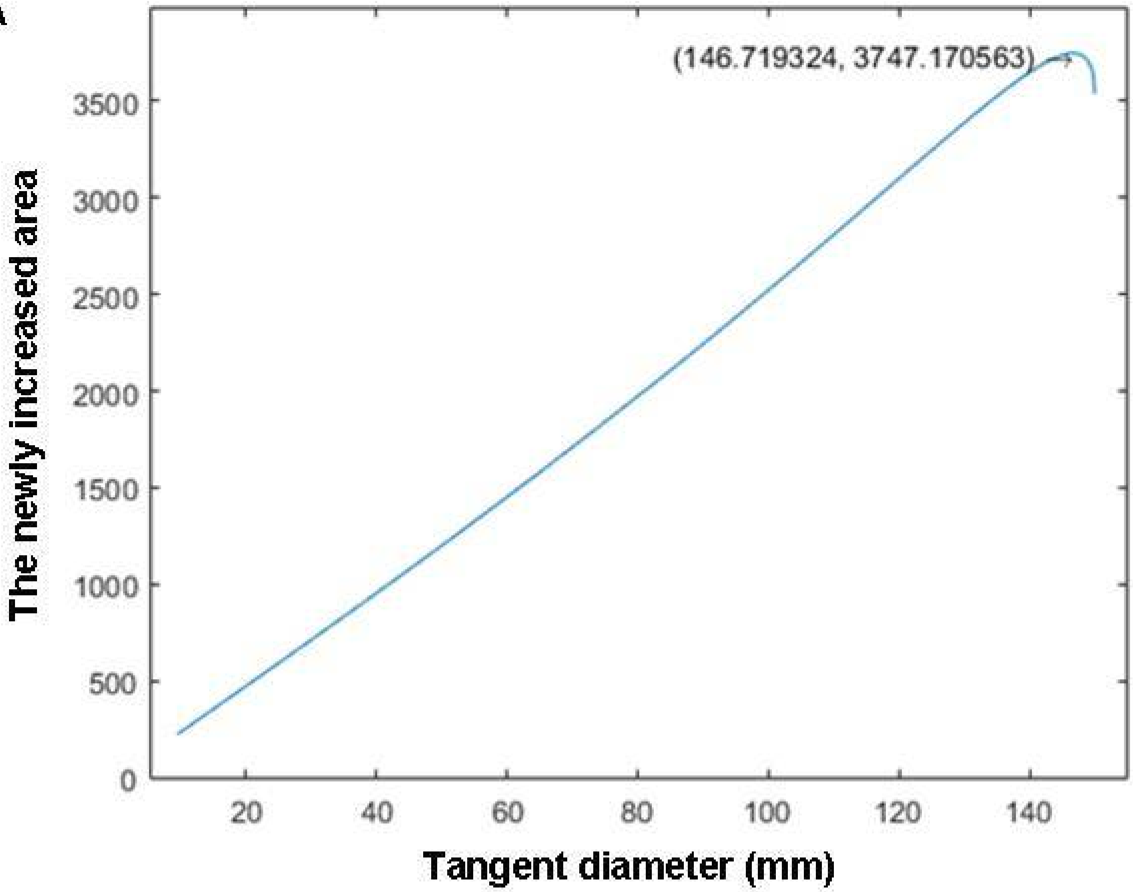

(146.719324, 3747.170563)
3500
3000
2500
2000
1500
1000
500
0
20
40
60
80
100
120
140
The newly increased area
Tangent diameter (mm)

B

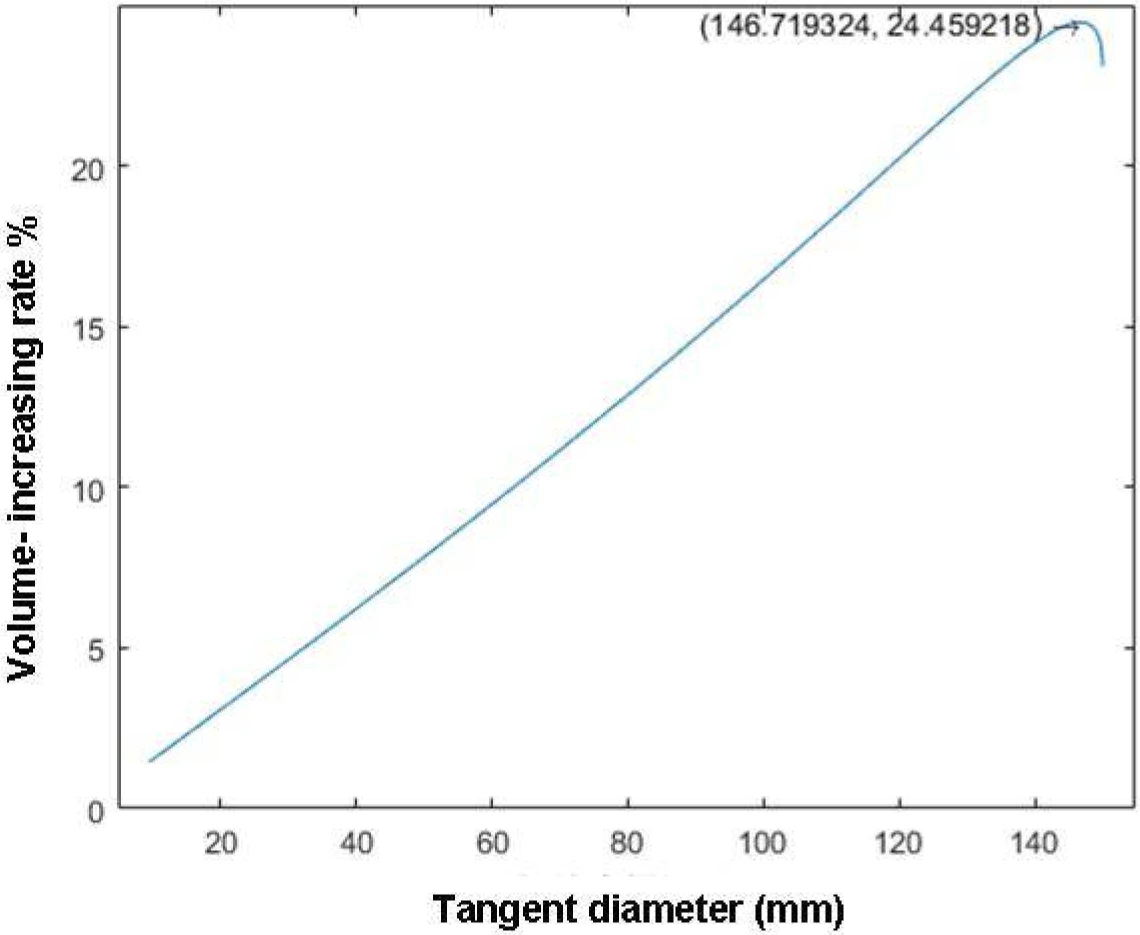

(146.719324, 24.459218)
20
15
10
5
0
20
40
60
80
100
120
140
Volume- increasing rate %
Tangent diameter (mm)

Supplementary Fig.S4. Plots of numerical integration for volume (A) and volume-increasing rate (B) in the proposed ellipse model with various tangent diameters (a=65, b=75, K=40mm).

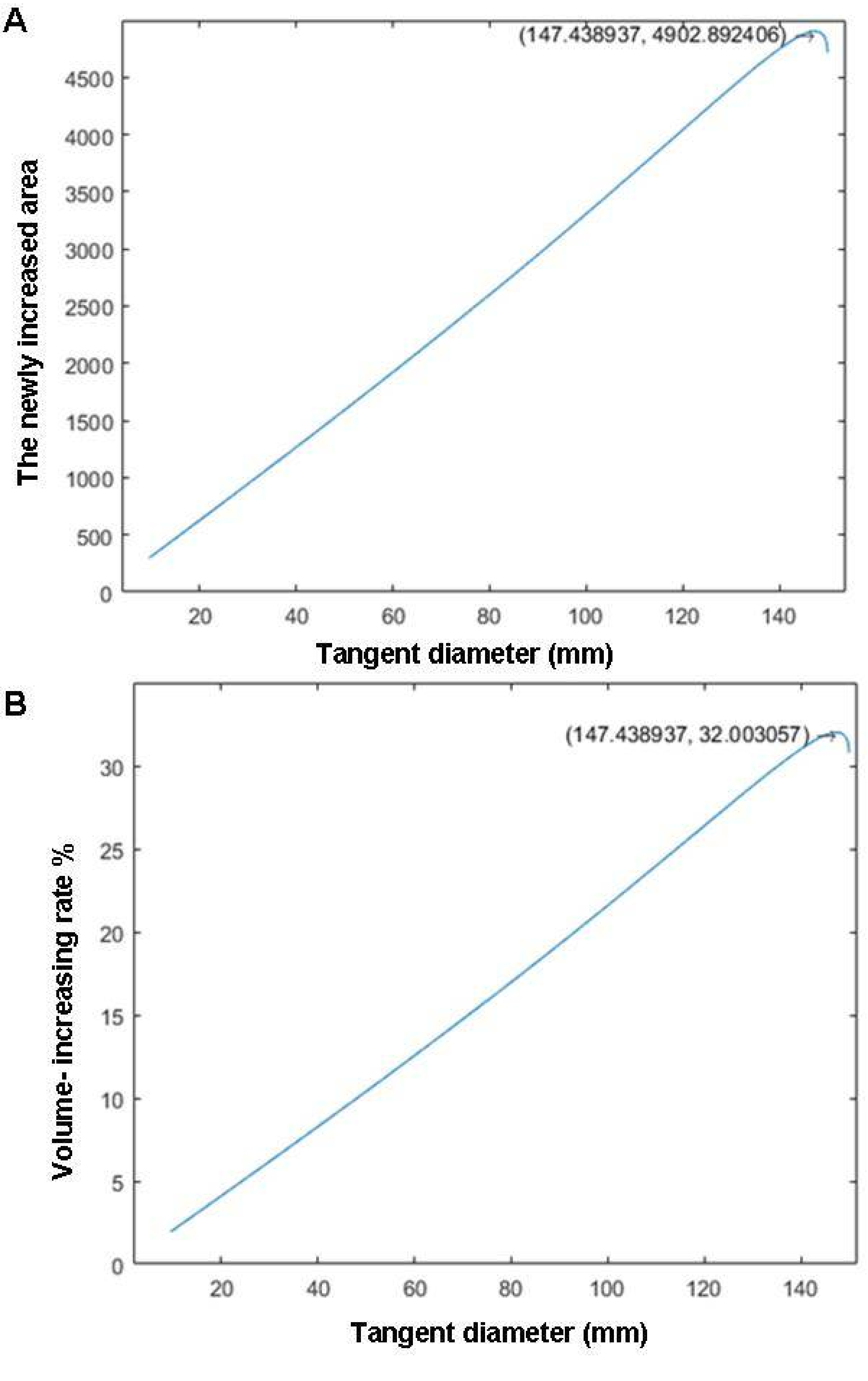
A
(147.438937, 4902.892406)
The newly increased area
Tangent diameter (mm)
B
(147.438937, 32.003057)
Volume- increasing rate %
Tangent diameter (mm)

Supplementary Fig.S5. Plots of numerical integration for volume (A) and volume-increasing rate (B) in the proposed ellipse model with various tangent diameters (a=65, b=75, K=50mm).

A

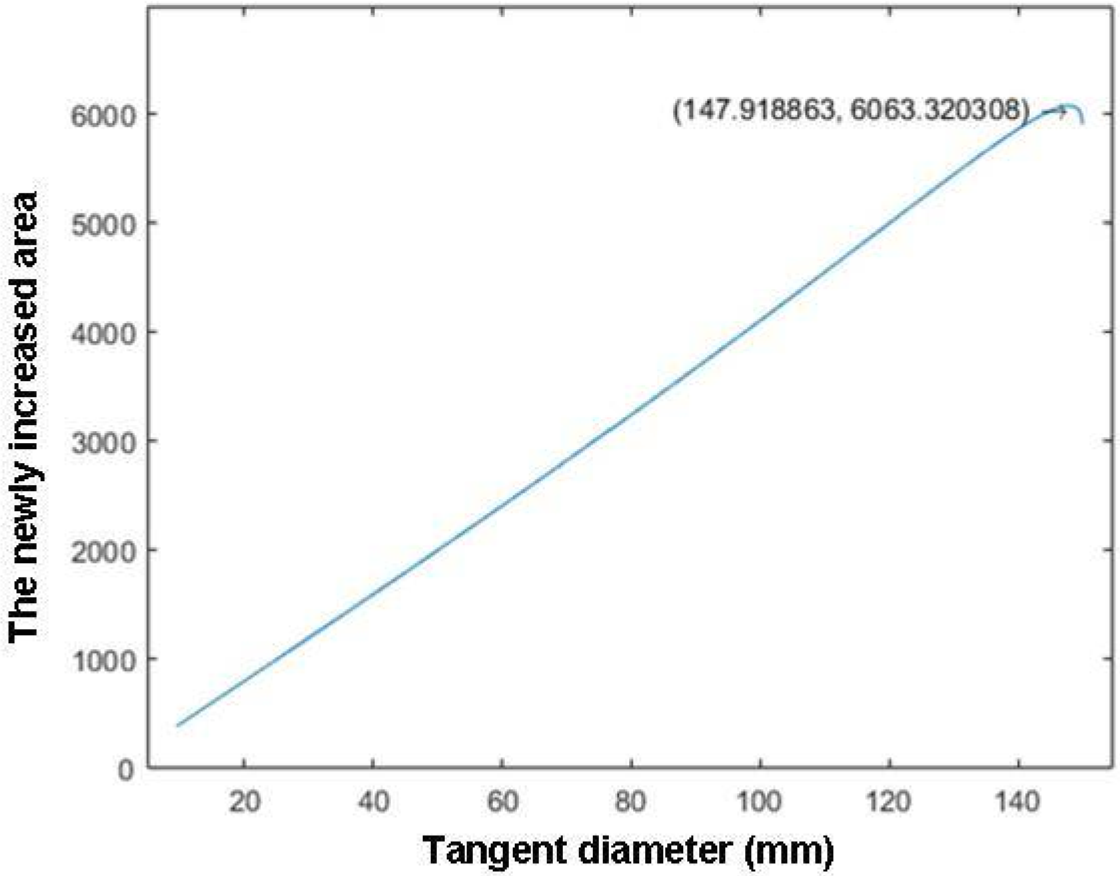
(147.918863, 6063.320308)
6000
5000
4000
3000
2000
1000
0
20
40
60
80
100
120
140
The newly increased area
Tangent diameter (mm)

B

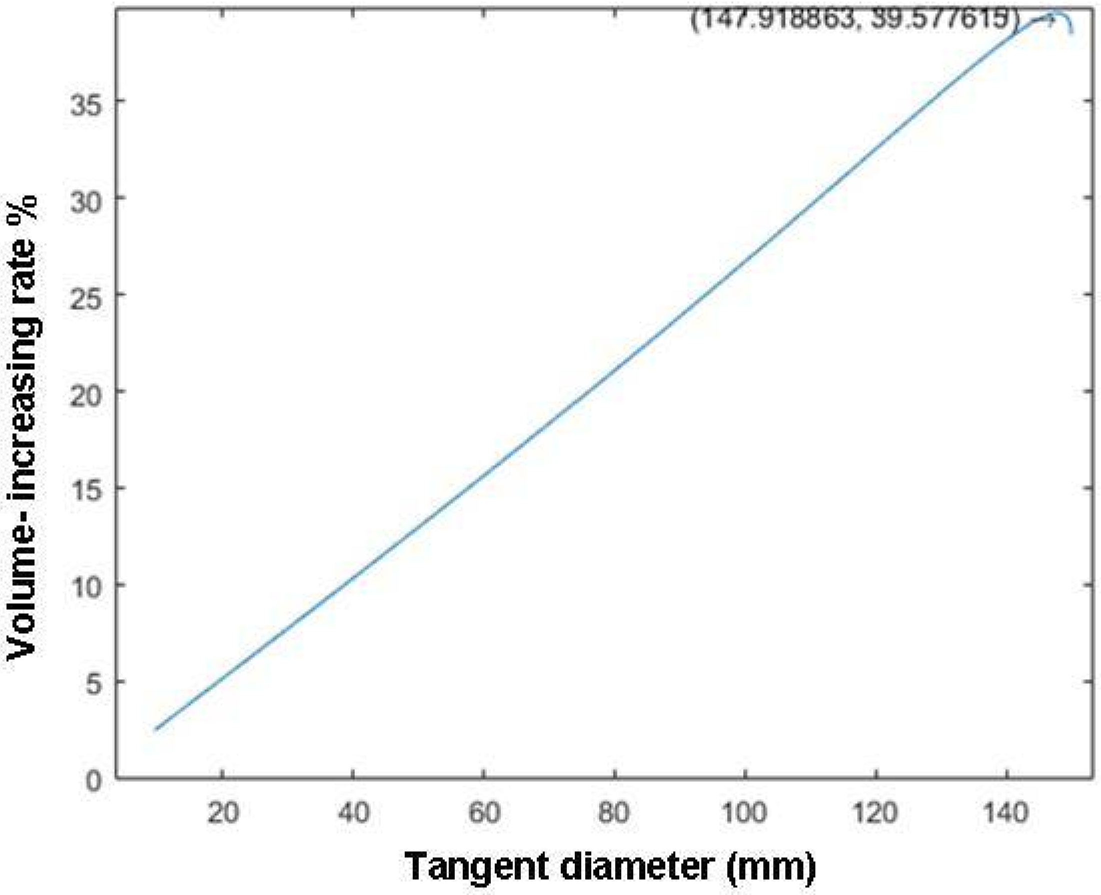
(147.918863, 39.577615)
35
30
25
20
15
10
5
0
20
40
60
80
100
120
140
Volume- increasing rate %
Tangent diameter (mm)

Supplementary Text S1

Results for volume in the proposed ellipse model with various tangent diameters
(a= 65, b=75, K=10mm)

For 500 consecutive data of different tangential diameters, first, plateau, maximum value (150), and respective previous data are retained; others are omitted.

The plateau value is shown in italics.

Tangential diameter = 9.482088 (mm) The newly increased area = 74.642045

:

Tangential diameter = 143.462748 (mm) The newly increased area = 1461.613563
Tangential diameter = 143.553997 (mm) The newly increased area = 1461.631628

:

Tangential diameter = 149.999700 (mm) The newly increased area = 1183.695994
Tangential diameter = 150.000000 (mm) The newly increased area = 1179.530315

Supplementary Text S2

Results for volume-increasing rate in the proposed ellipse model with various tangent diameters (a= 65, b=75, K=10mm)

The plateau value is shown in italics.

Tangential diameter = 9.482088 (mm) Volume increasing rate = 0.487217

:

Tangential diameter = 143.462748 (mm) Volume increasing rate = 9.540512
Tangential diameter = 143.553997 (mm) Volume increasing rate = 9.540630

:

Tangential diameter = 149.999700 (mm) Volume increasing rate = 7.726437
Tangential diameter = 150.000000 (mm) Volume increasing rate = 7.699246

Supplementary Text S3

Results for volume in the proposed ellipse model with various tangent diameters (a= 65, b=75, K=20mm)

The plateau value is shown in italics.

Tangential diameter = 9.482088 (mm)   The newly increased area = 149.137588

:

Tangential diameter = 145.466285 (mm)   The newly increased area = 2598.503638
Tangential diameter = 145.541437 (mm)   The newly increased area = 2598.532080

:

Tangential diameter = 149.999700 (mm)   The newly increased area = 2362.438388
Tangential diameter = 150.000000 (mm)   The newly increased area = 2358.275547

Supplementary Text S4

Results for volume-increasing rate in the proposed ellipse model with various tangent diameters (a= 65, b=75, K=20mm)

The plateau value is shown in italics.

Tangential diameter = 9.482088 (mm)   Volume increasing rate = 0.973478

:

Tangential diameter = 145.466285 (mm)   Volume increasing rate = 16.961429
Tangential diameter = 145.541437 (mm)   Volume increasing rate = 16.961615

:

Tangential diameter = 149.999700 (mm)   Volume increasing rate = 15.420541
Tangential diameter = 150.000000 (mm)   Volume increasing rate = 15.393368

Supplementary Text S5

Results for volume in the proposed ellipse model with various tangent diameters (a= 65, b=75, K=30mm)

The plateau value is shown in italics.

Tangential diameter = 9.482088 (mm)   The newly increased area = 223.633129

:

Tangential diameter = 146.655208 (mm)   The newly increased area = 3747.159739
Tangential diameter = 146.719324 (mm)   The newly increased area = 3747.170563

:

Tangential diameter = 149.999700 (mm)   The newly increased area = 3541.073595
Tangential diameter = 150.000000 (mm)   The newly increased area = 3536.913444

Supplementary Text S6

Results for volume-increasing rate in the proposed ellipse model with various tangent diameters (a= 65, b=75, K=30mm)

The plateau value is shown in italics.

Tangential diameter = 9.482088 (mm)   Volume increasing rate = 1.459739

:

Tangential diameter = 146.655208 (mm)   Volume increasing rate = 24.459147
Tangential diameter = 146.719324 (mm)   Volume increasing rate = 24.459218

:

Tangential diameter = 149.999700 (mm)   Volume increasing rate = 23.113944
Tangential diameter = 150.000000 (mm)   Volume increasing rate = 23.086789

Supplementary Text S7

Results for volume in the proposed ellipse model with various tangent diameters (a= 65, b=75, K=40mm)

The plateau value is shown in italics.

Tangential diameter = 9.482088 (mm) The newly increased area = 298.128671

:

Tangential diameter = 147.382462 (mm) The newly increased area = 4902.877304
Tangential diameter = 147.438937 (mm) The newly increased area = 4902.892406

:

Tangential diameter = 149.999700 (mm) The newly increased area = 4719.658661
Tangential diameter = 150.000000 (mm) The newly increased area = 4715.501123

Supplementary Text S8

Results for volume-increasing rate in the proposed ellipse model with various tangent diameters (a= 65, b=75, K=40mm)

The plateau value is shown in italics.

Tangential diameter = 9.482088 (mm) Volume increasing rate = 1.946000

:

Tangential diameter = 147.382462 (mm) Volume increasing rate = 32.002959
Tangential diameter = 147.438937 (mm) Volume increasing rate = 32.003057

:

Tangential diameter = 149.999700 (mm) Volume increasing rate = 30.807020
Tangential diameter = 150.000000 (mm) Volume increasing rate = 30.779883

Supplementary Text S9

Results for volume in the proposed ellipse model with various tangent diameters
(a= 65, b=75, K=50mm)

The plateau value is shown in italics.

Tangential diameter = 9.482088 (mm) The newly increased area = 372.624213

:

Tangential diameter = 147.868049 (mm) The newly increased area = 6063.286044
Tangential diameter = 147.918863 (mm) The newly increased area = 6063.320308

:

Tangential diameter = 149.999700 (mm) The newly increased area = 5898.214523
Tangential diameter = 150.000000 (mm) The newly increased area = 5894.059548

Supplementary Text S10

Results for volume-increasing rate in the proposed ellipse model with various tangent diameters (a= 65, b=75, K=50mm)

The plateau value is shown in italics.

Tangential diameter = 9.482088 (mm) Volume increasing rate = 2.432261

:

Tangential diameter = 147.868049 (mm) Volume increasing rate = 39.577391
Tangential diameter = 147.918863 (mm) Volume increasing rate = 39.577615

:

Tangential diameter = 149.999700 (mm) Volume increasing rate = 38.499906
Tangential diameter = 150.000000 (mm) Volume increasing rate = 38.472785
